# Comprehensive mutant chemotyping reveals embedding of a lineage-specific biosynthetic gene cluster in wider plant metabolism

**DOI:** 10.1073/pnas.2417588122

**Published:** 2025-03-19

**Authors:** Xue Qiao, Alan Houghton, James Reed, Burkhard Steuernagel, Jiahe Zhang, Charlotte Owen, Aymeric Leveau, Anastasia Orme, Thomas Louveau, Rachel Melton, Brande B. H. Wulff, Anne Osbourn

**Affiliations:** ^a^Department of Biochemistry and Metabolism, John Innes Centre, Norwich NR4 7UH, United Kingdom; ^b^State Key Laboratory of Natural and Biomimetic Drugs, School of Pharmaceutical Sciences, Peking University, Beijing 100191, China; ^c^Department of Computational and Systems Biology, John Innes Centre, Norwich NR4 7UH, United Kingdom; ^d^Department of Crop Genetics, John Innes Centre, Norwich NR4 7UH, United Kingdom

**Keywords:** metabolism, biosynthesis, precursor supply genes, noncanonical enzymes, acyl sugars

## Abstract

Plants produce diverse specialized metabolites with important ecological functions. It has recently become apparent that the genes for many of these pathways are not dispersed in plant genomes, but rather are arranged like beads on a string in biosynthetic gene clusters (BGCs). Pathways encoded by BGCs are as a rule dedicated linear pathways that do not form parts of wider metabolic networks. In contrast, the genes for the biosynthesis of widely distributed more ancestral metabolites such as carotenoids and anthocyanins are not clustered. Little is known about how these more recently evolved clustered pathways interact with general plant metabolism. We recently characterized a 12-gene BGC for the biosynthesis of the antimicrobial defense compound avenacin A-1, a triterpene glycoside produced by oats. Avenacin A-1 is acylated with the fluorophore *N*-methyl anthranilate and confers bright blue fluorescence of oat root tips under ultraviolet light. Here, we exploit a suite of >100 avenacin-deficient mutants identified by screening for reduced root fluorescence to identify genes required for the function of this paradigm BGC. Using a combination of mutant chemotyping, biochemical and molecular analysis, and genome resequencing, we identify two nonclustered genes (*Sad4* and *Pal2*) encoding enzymes that synthesize the donors required for avenacin glycosylation and acylation (recruited from the phenylpropanoid and tryptophan pathways). Our finding of these Cluster Auxiliary Enzymes (CAEs) provides insights into the interplay between general plant metabolism and a newly evolved lineage-specific BGC.

## Significance

The ability of plants to biosynthesize particular types of specialized metabolites is commonly restricted to narrow taxonomic groups and is most likely a reflection of niche adaptation. Over 40 plant biosynthetic gene clusters (BGCs) have now been reported. However, our understanding of how these BGCs draw on and interact with general plant metabolism is very limited. Here, using a paradigm BGC as our exemplar (the oat avenacin cluster), we establish a comprehensive mutant chemotyping approach and identify two genes (*Sad4* and *Pal2*) that contribute to avenacin biosynthesis by furnishing acyl and sugar donors required for tailoring. Our investigation provides an exemplar for identifying genes that facilitate BGC function and sheds light on the interplay between specialized and general metabolism.

Plants produce a huge array of specialized metabolites (also referred to as natural products) with important ecological functions (1, 2). The evolution of the ability to biosynthesize particular types of specialized metabolites is commonly restricted to specific plant lineages, and is likely a reflection of adaptation to different environmental niches (3). Considerable progress has been made in recent years in elucidating the genetic basis of numerous plant pathways for the biosynthesis of specialized plant metabolites (4–6). As the body of available genome sequences has grown, it has become clear that the organization of biosynthetic pathway genes in genomes in physically linked clusters is a common phenomenon across the Plant Kingdom. Plant Biosynthetic Gene Clusters (BGCs) typically span tens to hundreds of kilobases and contain multiple physically colocalized nonhomologous genes encoding the enzymes needed to catalyze the different reactions of a plant specialized metabolite pathway (7–12). The existence of these BGCs in plant genomes is intriguing. A first assumption may be that these “operon-like” BGCs have originated by horizontal gene transfer from microbes (12). However, there is no evidence to support this hypothesis from the >40 plant BGCs characterized to date (3). Rather these clustered pathways appear to have arisen by duplication and neofunctionalization of existing plant genes recruited from existing metabolism. The organization of the newly evolved genes into a BGC is presumably a consequence of selection pressure, since bringing the genes into physical proximity would facilitate coinheritance of beneficial combinations of alleles that together confer a selective advantage and mitigate against genetic disruption of the pathway (3, 11, 13).

Clustering appears to be a feature of biosynthetic pathways that have evolved relatively recently in evolutionary time in particular plant lineages (8–10). Other older pathways that are widely distributed across plants (e.g. for carotenoids and anthocyanins) tend not to be clustered. Nevertheless, BGCs will need to “plug into” these more conserved facets of metabolism in order to access precursors and cofactors (14). The process of BGC evolution and of how such clustered pathways are embedded in wider metabolism is not well understood.

**Fig. 1.**
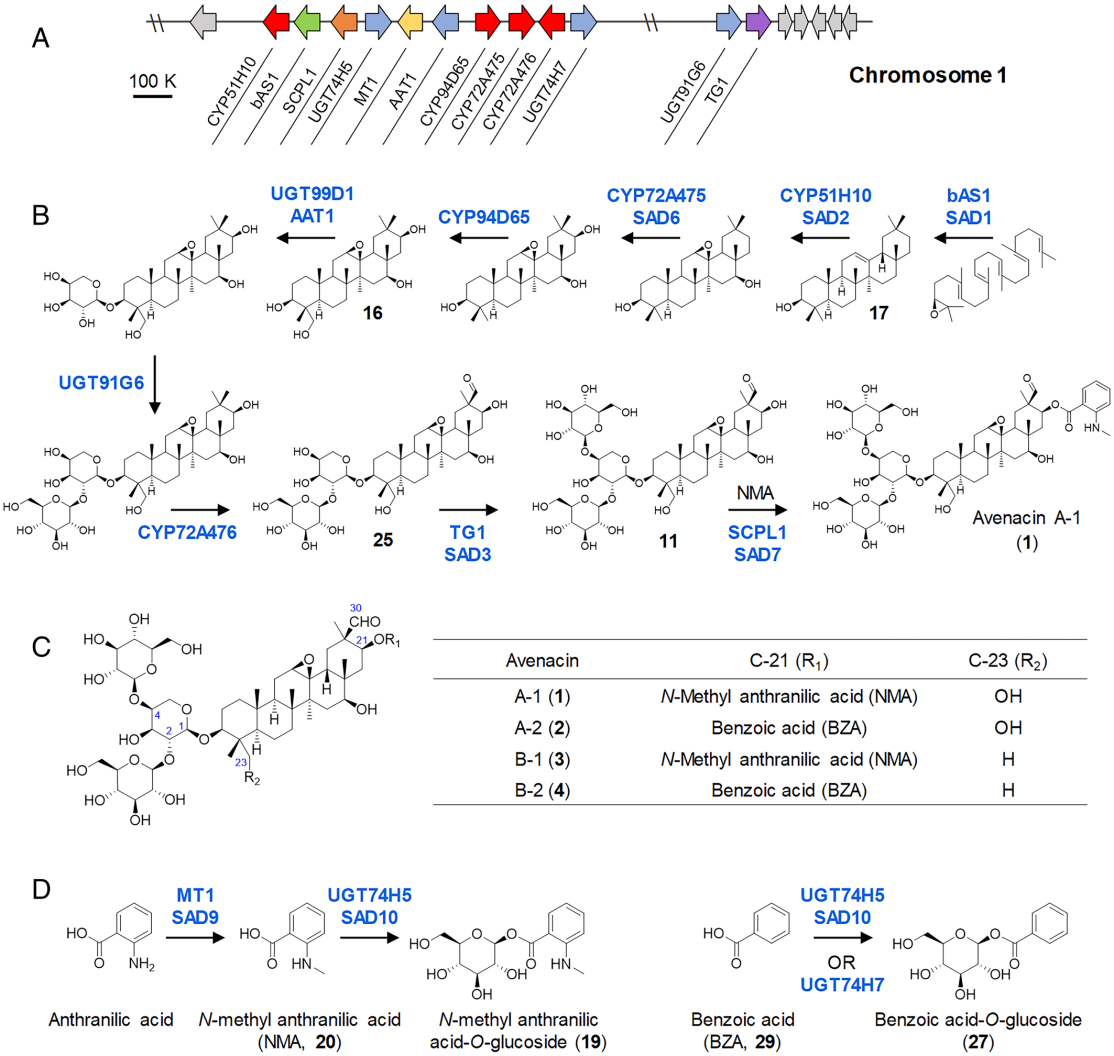
Structures and biosynthetic pathways of avenacins in oat. (*A*) The avenacin biosynthetic gene cluster. (*B*) Biosynthetic route for avenacin A-1. (*C*) Structures of the four oat avenacins. (*D*) Biosynthetic pathways of acyl sugars. Previously characterized pathway enzymes are indicated: bAS1/SAD1, β-amyrin synthase (16) CYP51H10/SAD2, C-12,13β epoxy, C-16β oxidase (17) CYP72A475/SAD6, C-21β oxidase (23) CYP94D65, C-23 oxidase (15) CYP72A476, C-30 oxidase (15) AAT1/UGT99D1, triterpene 3-*O*-arabinosyltransferase (22) UGT91G16, β-[1, 2]-glucosyltransferase (24) TG1/SAD3, transglucosidase (24) SCPL1/SAD7, serine carboxypeptidase-like acyltransferase (19) MT1/SAD9, anthranilate *N*-methyltransferase (20) UGT74H5/SAD10, *N*-methylanthranilic acid *O*-glucosyltransferase (21) UGT74H7, benzoic acid *O*-glucosyltransferase (21).

The pathway for the biosynthesis of antimicrobial defense compounds in oats (genus *Avena*) has emerged as a paradigm plant BGC (15). This cluster contains 12 contiguous genes that are necessary and sufficient for the biosynthesis of triterpene glycosides known as avenacins (Fig. 1*B*) (15–24). Avenacins are produced in the epidermal cells of the root tips and lateral root initials, where they provide protection against soil-borne pathogens (25). These compounds are only produced by *Avena* species and are not found in other cereals and grasses, leading to interest in the feasibility of engineering the avenacin pathway into crops such as wheat with the aim of enhancing disease resistance (25). The avenacin BGC is located in a subtelomeric region of the oat genome that lacks synteny with other members of the Gramineae (15).

Oats produce four structurally related avenacins, A-1, B-1, A-2, and B-2 (Fig. 1*C*). Avenacins A-1 and B-1 are acylated with an *N*-methyl anthranilic acid group at the carbon 21 position, while avenacins A-2 and B-2 have a benzoic acid group at this position instead. Avenacins A-1 and B-1 are autofluorescent under ultraviolet (UV) illumination because N-methyl anthranilate (NMA) is a fluorophore, while avenacins A-2 and B-2 have little/no fluorescence. The major avenacin found in oat roots is avenacin A-1, which is responsible for the bright blue fluorescence of oat root tips under UV light. We previously exploited the UV fluorescence of avenacin A-1 to carry out a forward genetic screen for avenacin-deficient mutants of diploid oat (*Avena strigosa* accession S75) following sodium azide mutagenesis. These mutants are known as *saponin-deficient* (*sad*) mutants (25).

The core avenacin scaffold is derived from the isoprenoid pathway by cyclization of 2,3-oxidosqualene, mediated by the oxidosqualene cyclase enzyme β-amyrin synthase (bAS1/SAD1) (16). This scaffold is then oxygenated, glycosylated, and acylated to give avenacin A-1 (Fig. 1*B*). The addition of the acyl group is carried out by the serine carboxypeptidase-like acyltransferase SAD7 (SCPL1) (19). Unlike BAHD acyltransferases, which use CoA-activated acyl donors, serine carboxypeptidase-like acyltransferases utilize acyl sugars as their donors. The *Sad7* (*SCPL1*) gene lies within the avenacin BGC next to genes encoding the methyltransferase *MT1* (*SAD9*) (20), and the UDP-dependent d-glucosyl transferase *UGT74H5* (*SAD10*) (21) (Fig. 1*A*), which together make NMA glucoside (19), the acyl sugar donor needed for the biosynthesis of avenacins A-1 and B-1 (Fig. 1*D*). These three genes therefore encode a trio of enzymes required for the biosynthesis and addition of the NMA acyl group (19–21).

In this study, to understand how clustered pathways are embedded in wider metabolism, we exploit the oat mutant collection that we have established to carry out systematic metabolite profiling. Through a combination of chemotyping, genetics, and genome resequencing, we identify two genes, *Sad4* and *Pal2*, associated with the general phenylpropanoid and tryptophan pathways. These genes are not located within the avenacin BGC but are essential for avenacin biosynthesis. Our evidence indicates that the enzymes encoded by *Sad4* and *Pal2* likely generate the precursors required for the last two steps in avenacin biosynthesis—addition of the acyl group by SAD7/SCP1 (19) and addition of the final sugar of the trisaccharide chain (a d-glucose) by the noncanonical sugar transferase SAD3/TG1 (24). These findings provide insights into our understanding of the interaction between this BGC-encoded specialized metabolic pathway and general metabolism.

## Results

### Characterization of Avenacin-Deficient Mutants.

**Fig. 2.**
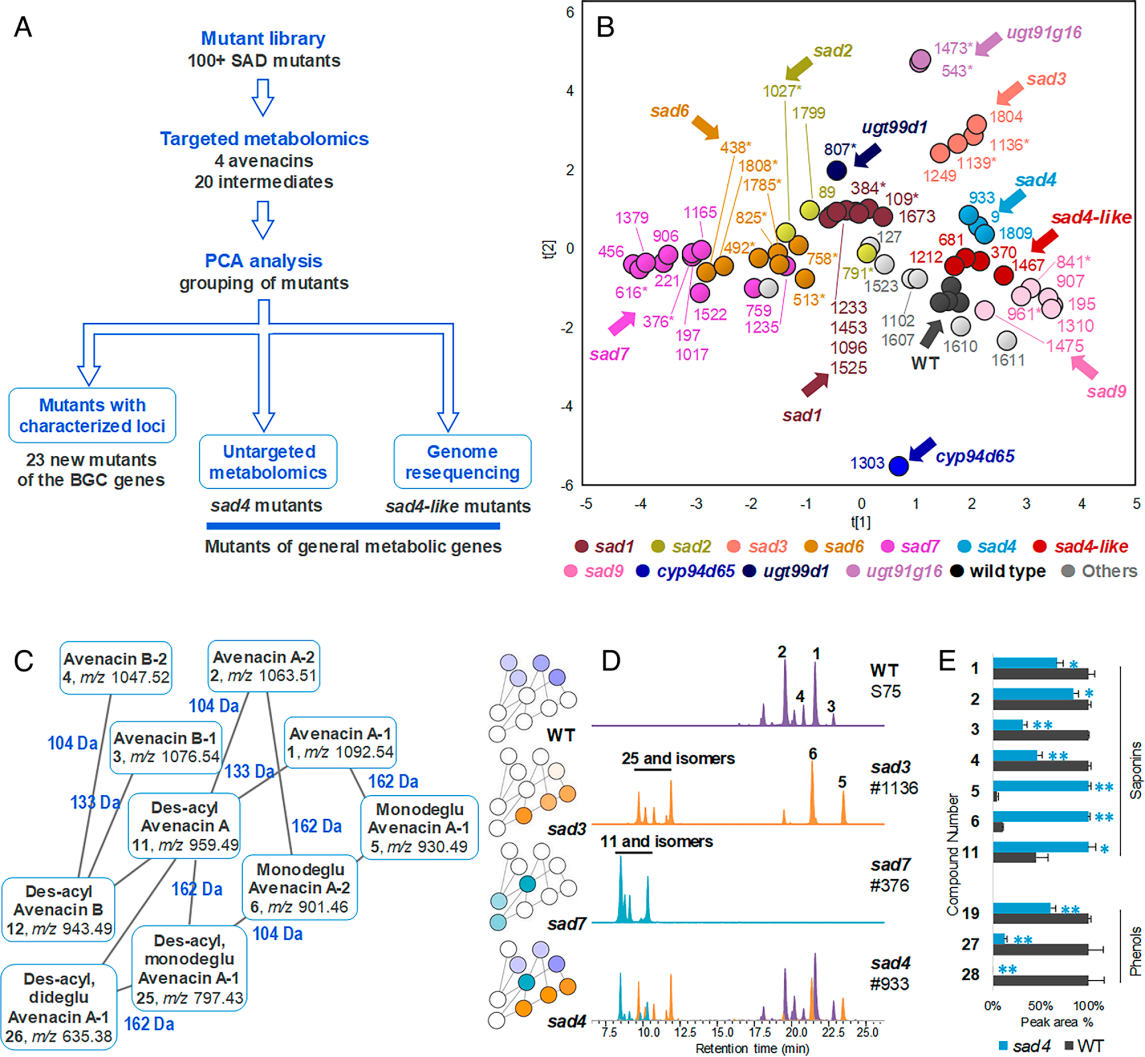
Chemical analysis of root extracts for avenacin-deficient oat mutants. (*A*) Workflow of comprehensive chemotyping to characterize the mutants. (*B*) Principal component analysis of targeted LC/MS data for different mutant lines. *, mutant lines identified previously; WT, wild-type oat. (*C*) Metabolic network analysis of LC/MS/MS data for WT oat and selected mutants using GNPS (26). (*D*) Extracted ion chromatogram for key metabolites of WT oat and selected mutants. All metabolites were extracted for the [M-H]^−^ ion shown in panel *C* in (−)-ESI-MS mode. *m*/*z* 959.49 and *m*/*z* 797.43 were zoomed in by a factor of 5. (*E*) Relative contents of saponins and phenols of the wild-type and *sad4* mutants. Peak areas (±SE) were from untargeted metabolomics (for WT, *n* = 3 biological replications; for *sad4* mutant, *n* = 3 different lines #9, #933, #1809). *P < 0.05, **P < 0.01 comparing to the wild type (Student’s *t* test). Metabolite identities: avenacin A-1 (**1**); avenacin A-2 (**2**); avenacin B-1 (**3**); avenacin B-2 (**4**); monodeglucosyl avenacin A-1 (**5**); monodeglucosyl avenacin A-2 (**6**); des-acyl avenacin A (**11**) and isomers; monodeglucosyl avenacin B (**12**); des-acyl monodeglucosyl avenacin A (**25**) and isomers; des-acyl, dideglucosyl avenacin A (**26**); *N*-methyl anthranilic acid *O*-glucoside (**19**), benzoic acid *O*-glucoside (**27**), and ferulic acid *O*-glucoside (**28**).

The fluorescence-based screen that we used to identify avenacin-deficient mutants of diploid oat (*A. strigosa*) was highly sensitive, enabling us to isolate around 100 mutants with altered avenacin content (25). This comprehensive mutant resource opens up an opportunity to investigate BGC function. By employing targeted metabolomic analysis, it is possible to swiftly pinpoint mutants for previously characterized BGC genes. Through the integration of untargeted metabolomic analysis with mass spectrometry (MS) network analysis, the metabolic characteristics of mutants with uncharacterized loci can be elucidated, and the disrupted metabolic pathways and genes can be traced (Fig. 2*A*).

Previously, we have shown that a total of 55 of these mutants correspond to eight of the characterized avenacin BGC genes [(15–25, 27), *SI Appendix,* Table S1]. Mutants for the remaining four characterized genes [*CYP94D65*, *CYP72A476*, *Sad10/UGT74H5*, and *UGT74H7* (15, 21)] have not as yet been identified. We carried out liquid chromatography/mass spectrometry (LC/MS) targeted metabolomics of root extracts of a further 38 uncharacterized mutants, including mutant lines representing previously cloned and characterized loci for reference (Fig. 2*B*). The peak areas of the four avenacins, 16 known pathway intermediates and four proposed intermediates (**1**–**24**, *SI Appendix,* Fig. S1) were determined and analyzed using principal component analysis. A total of 23 of the new mutants grouped with mutants representing previously characterized loci, including *bAS1/Sad1* (5 new mutants), *CYP51H10/Sad2* (2 new mutants), *SCPL1*/*Sad7* (10 new mutants), *MT1*/*Sad9* (4 new mutants), and *TG1/Sad3* (2 new mutants) (*SI Appendix,* Figs. S2–S4). Single nucleotide variants (SNVs) in the coding sequences of the cognate genes were confirmed by PCR amplification and sequencing. These mutants therefore represent new alleles of previously characterized avenacin pathway genes (*SI Appendix,* Table S1). A further mutant, #1303, had a clearly distinct metabolite profile compared to all of the other mutant lines (Fig. 2*B*). LC/MS analysis showed that the predominant avenacins in WT root extracts were A-1 and A-2, as expected (25). In contrast, the avenacin content of mutant #1303 was shifted to primarily B-1 and B-2, while A-1 and A-2 were not detectable (*SI Appendix,* Fig. S5). Since avenacins A-1 and A-2 are hydroxylated at the C-23 position while avenacins B-1 and B-2 are not, this chemical profile suggests a defect in C-23 oxidation. We recently identified a cytochrome P450 enzyme CYP94D65 that is able to add a hydroxyl group to the β-amyrin scaffold at this position (15). The *CYP94D65* gene is located in the avenacin biosynthetic gene cluster and was originally characterized by transient expression in *Nicotiana benthamiana*. Corresponding mutants had not been identified previously (15). DNA sequence analysis of the *CYP94D65* gene in mutant #1303 revealed a mutation (G1385A) predicted to cause premature termination of translation (*SI Appendix,* Table S1).

### Metabolic Profiling of sad4 and sad4-Like Mutants.

The remaining 14 mutants had metabolite profiles that were distinct from the other mutants. Two other groups of mutants clustered close to the WT in Fig. 2*B*. One of these groups included two mutants (#9 and #933) that we had previously shown correspond to an unlinked and as yet uncloned locus (*Sad4*) required for avenacin glycosylation and normal root growth (18), and a third mutant (#1809). The similarities between #1809 and the two genetically confirmed *sad4* mutants suggest that this line represents another mutant allele of *Sad4*. *sad4* mutants have reduced levels of avenacins A-1 (**1**) and A-2 (**2**) and, unlike the wild type, accumulate detectable levels of monodeglucosyl avenacin A-1 (**5**) and A-2 (**5**) (18). The second group of mutants (#370, 681, 1212, 1467) also had reduced levels of A-2 (**2**) and accumulated monodeglucosyl avenacins A-1 (**5**) and A-2 (**6**), but were unaffected in avenacin A-1 (**1**) levels (*SI Appendix,* Fig. S6). We named this second group of mutants *sad4-like.* We then carried out in-depth comparisons of the *sad4* and *sad4-like* mutants using untargeted metabolomics for extracts from both roots and shoots of 12-d-old plants. The top 240 marker peaks that distinguished *sad4* and *sad4-like* mutants from the wild type were selected and their relative peak areas presented in a heat map (*SI Appendix,* Fig. S7). This revealed that the *sad4* and *sad4-like* mutations affect both the roots and shoots, with *sad4* mutants showing the most marked effect.

To investigate the chemotype of *sad4* mutants further, untargeted metabolomics data were generated for the wild type and for *sad4* (#9, 933, 1809), *sad3* (#1136, 1429, 1804), and *sad7* (#1379, 376, 616) mutants and analyzed using the Global Natural Products Social Molecular Networking server (GNPS, https://gnps.ucsd.edu/). A simplified mass spectral network (Fig. 2*C*) showed that the key chemical markers for *sad4* mutants are primarily avenacin intermediates lacking 162 Da, 133 Da, and 104 Da groups. By analyzing the mass spectra, these fragments were assigned as glucosyl-, *N*-methyl anthraniloyl-, and benzoyl- groups. We have previously cloned and characterized the enzymes that attach these groups: SAD3/TG1 is a transglucosidase that catalyzes the last glycosylation step (the addition of the 1,4-linked d-glucose to the sugar chain at the C-3 position of the triterpene scaffold) (24), while SAD7/SCPL1 is a serine carboxypeptidase-like acyltransferase that adds the acyl group (*N* -methyl anthranilic acid or benzoic acid) at the C-21 position (19). Further metabolite analysis revealed that *sad4* mutants have a “hybrid” chemical profile relative to the wild type, *sad3* and *sad7* lines (Fig. 2 *C* and *D*): Root extracts of *sad4* mutants contain the major intermediates observed in *sad3* and *sad7* lines as well as the pathway end products, avenacins.

### Identification, Cloning, and Functional Characterization of *Sad4*.

Root extracts of *sad4* mutants have reduced levels of avenacins compared to the WT, and increased levels of deglucosylated- and des-acyl-avenacins (**5**, **6**, **11**), the precursors for SAD3/TG1 and SAD7/SCPL1, respectively (Fig. 2*E*). The acyl donor for the serine carboxypeptidase-like acyl transferase SAD7/SCPL1 is anticipated to be an acyl sugar—either *N*-methyl anthranilic acid glucoside (**19**) (avenacins A-1 and B-1) or benzoic acid glucoside (**27**) (avenacins A-2 and B-2) (19). The sugar donor for the noncanonical glucosyl transferase SAD3/TG1 (a transglucosidase), which adds the last sugar to the trisaccharide sugar chain of the avenacins, is also anticipated to be an acyl sugar (of d-glucose), such as **19**, **27,** or **28** (ferulic acid *O*-glucoside) (24). The levels of all three acyl sugars (**19**, **27**, **28**) are significantly decreased to varying extents in *sad4* mutants (Fig. 2*E* and *SI Appendix,* Fig. S8). The enzymes needed to make these donors are anticipated to be either glycosyltransferases or enzymes required for synthesis of the phenol scaffolds (28). *N*-methyl anthranilic acid is synthesized *via* the tryptophan pathway, while benzoic acid and ferulic acid are formed *via* the phenylpropanoid pathway (28). It is unlikely that *Sad4* is a phenol scaffold-building enzyme since the levels of all three acyl sugars are affected. We therefore hypothesized that *Sad4* may encode a sugar transferase involved in acyl sugar biosynthesis.

**Fig. 3.**
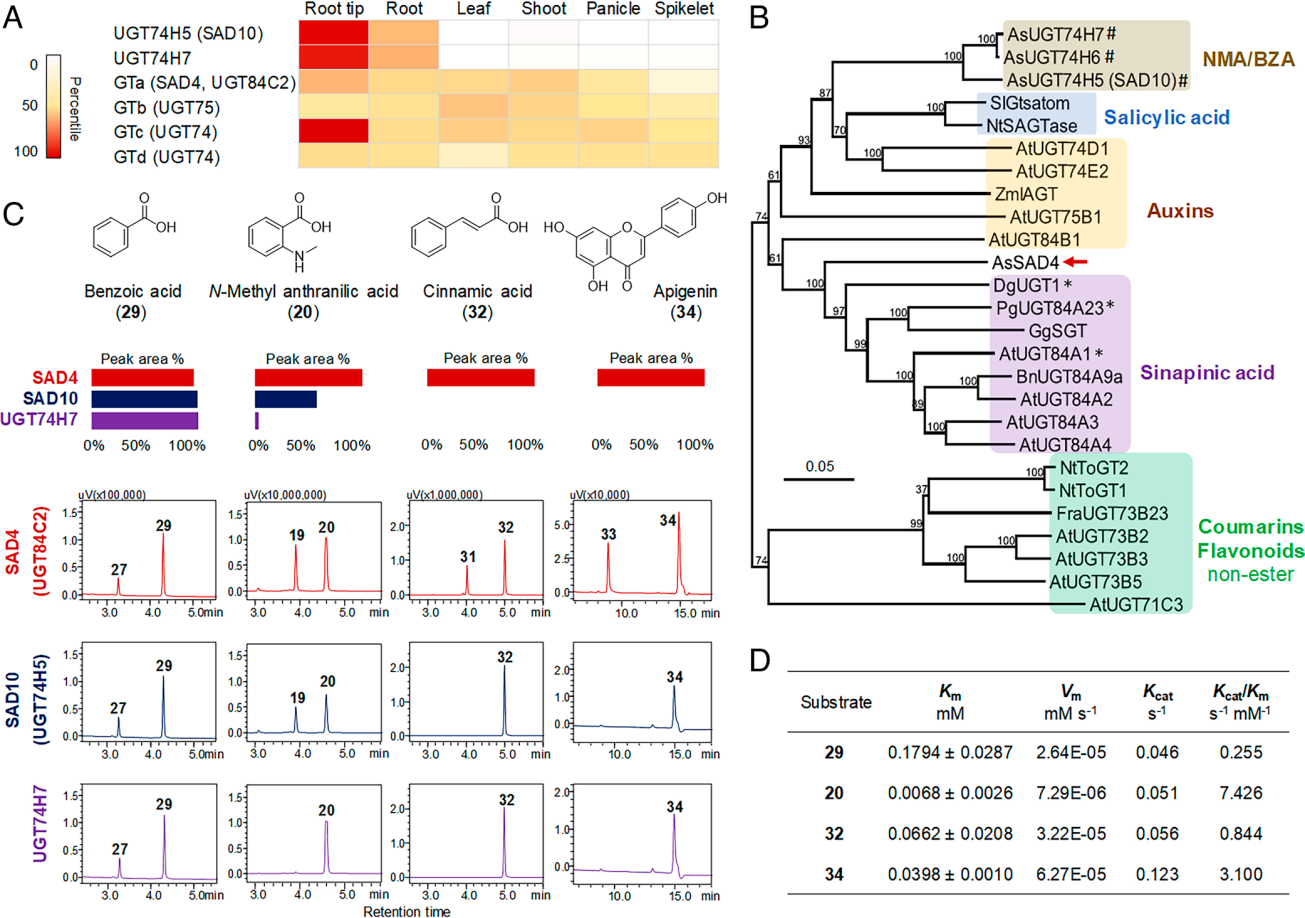
Expression pattern, phylogenetic analysis, and functional analysis for *Sad4*. (*A*) Expression pattern of previous characterized cluster phenol glucosyltransferases (21), candidates of *Sad4* locus, and UGT84 family genes in *A. strigosa*. (*B*) Phylogenetic analysis of SAD4 and previously characterized phenol glucosyltransferases. *, enzymes promiscuous to different substrates. For accession numbers, *SI Appendix,* Table S4. (*C*) Functional analysis of SAD4, SAD10, and UGT74H7 on four representative substrates. (*D*) Kinetic parameters for SAD4 using different substrates (means ± SD, *n* = 3).

The class of enzymes typically involved in plant natural product glycosylation are UDP-dependent glycosyltransferases (UGTs) belonging to glycosyl transferase family 1 (GT1) (29). We therefore mined the available RNA-seq resource for *A. strigosa* (15) for all predicted GT1 sequences using representative amino acid sequences of characterized family 1 UGTs (30) as query sequences (*SI Appendix,* Fig. S9*A* and Table S2). From the total of 173 GT1 hits, phylogenetic analysis revealed that 30 belong to Group L, several members of which have previously been shown to catalyze the formation of glucose ester bonds by recognizing carboxylic groups on a variety of different metabolites(31, 32). Since mutations at *Sad4* affect the chemical profiles of both the roots and the shoots (*SI Appendix,* Fig. S7), as well as the morphology of root tip (18), we speculated that the gene may be expressed in all three tissue types. The expression patterns of the 30 Group L UGT genes are shown in *SI Appendix,* Fig. S9*B*. Four predicted glycosyltransferases GTa-GTd (*AS01G000200*, *AS01G094620*, *AS05G011850*, *AS03G099380*) with absolute read counts of >100 in all of these tissue types were identified (Fig. 3*A*). These genes were amplified and sequenced in the three *sad4* mutants. Individual SNVs were identified in the GTa gene (*AS01G000200*) in all three mutants (#9, 933, 1809, Table 1). In each case, the amino acid changes were predicted using the SIFT algorithm (33) to affect protein function. SNVs were not found in the other three candidate GT1 genes.

**Table 1.** Characterization of *sad4* and *sad4-like* (*pal2*) mutants

Gene	Mutant	Mutation event	Amino acid change
*Sad4*	#9	C404T	P135L
	#933	G963A	W321 stop
	#1809	C404T	P135L
*Sad4-like*	#370	G323A	G108E
(*Pal2*)	#681	C451T	R151C
	#1212	G409A	G137R
	#1467	C556T	P186S

To confirm the identity of *Sad4*, 192 F_2_ progeny obtained from a cross between the wild type *A. strigosa* line S75 and the *sad4* mutant #933 were chemotyped and genotyped. This analysis confirmed that accumulation of monodeglucosyl avenacin A-1 (**5**) occurred specifically in lines that were homozygous mutant for *AS01G000200.* The segregation pattern was consistent with a 1:2:1 ratio (P > 0.9), as expected for a single dominant Mendelian gene (*SI Appendix,* Table S3). Collectively these data indicate that the GT1 gene *AS01G000200* corresponds to the previously uncharacterized *Sad4* locus (hereafter referred to as UGT84C2) (21). Phylogenetic analysis reveals that SAD4/UGT84C2 does not cluster with the sugar transferases encoded by previously characterized avenacin BGC genes *SAD10/UGT74H5* and *UGT74H7*, which are involved in the generation of the *N*-methyl anthranilic acid and benzoic acid acyl glucoside acyl donors, respectively (21) (Fig. 3*B* and *SI Appendix,* Table S4). Of note, three phylogenetically related GT1s (AtUGT84A1, PgUGT84A23, DgUGT1) within the UGT84 family (Fig. 3*B*) have been previously reported to accept various phenolic acids and flavonoids as substrates (34–36). Functional analysis of SAD4/UGT84C2 in vitro revealed that the enzyme is able to catalyze the addition of d-glucose to *N*-methyl anthranilic acid (**20**) and benzoic acid (**29**), so yielding potential acyl donors for SAD7/SCPL1 and glucosyl donors for SAD3/TG1 (Fig. 3*C*) (21). The function of SAD4/UGT84C2 therefore appears to overlap with those of other ester-forming GTs encoded by the avenacin BGC, SAD10/UGT74H5, and UGT74H7. However, evaluation of a wider range of compounds revealed that the substrate spectrum of SAD4/UGT84C2 is much broader when compared to SAD10/UGT74H5 and UGT74H7. SAD4/UGT84C2 is able to glucosylate at least 19 phenolic substrates, forming ester or aliphatic C-O bonds (*SI Appendix,* Table S5 and Figs. S10 and S11). Compared to the substrate spectrum of previous reported UGT84 family GTs, SAD4 is one of the family members with the broadest substrate spectrum (34–37). Kinetic analysis using four representative substrates revealed that *N*-methyl anthranilic acid (**20**) was preferred, followed by apigenin (**34**), cinnamic acid (**32**), and then benzoic acid (**29**) (Fig. 3 *C* and *D*). The glycosylated product for **34** was purified and characterized as its 7-*O* glucoside (**33**) (*SI Appendix*, *Supplement* 1). Interestingly, these substrates were from both the phenylpropanoid pathway (e.g. **29**, **32**, **34**) and the tryptophan pathway (e.g. **20**, anthranilic acid and indole-3-butyric acid). However, SAD4 was unable to glucosylate the substrate of SAD3/TG1 (24) (*SI Appendix,* Fig. S12). Expression of SAD4 in *N. benthamiana* boosted the production of benzoic acid *O*-glucoside (**27**), ferulic acid *O*-glucoside (**28**), and coumaric acid *O*-glucoside (**38**), compounds that were identical to the in vitro enzymatic reaction products of SAD4 (*SI Appendix,* Fig. S13). The glucoside of **20** was not detected in *N. benthamiana* leaves, likely due to the absence of the substrate. Given that *Sad4* is expressed in both the roots and the aerial tissues of oat (Fig. 3*A*) and the metabolic profiles of both roots and shoots, including for the foliar steroidal saponins, avenacosides, are altered in *sad4* mutants (*SI Appendix*, Figs. S7 and S14), SAD4/UGT84C2 appears to be a broad spectrum GT with multiple roles in metabolism.

### Identification, Cloning, and Functional Characterization of the *Sad4-Like* Gene.

To elucidate the function of the *Sad4-like* gene we used a genome resequencing approach. We previously generated a pseudochromosome-level assembly of the 4.1 Gb genome of *A. strigosa* accession S75, the line used in our original sodium azide mutagenesis experiment (15). We first tested our genome resequencing method using the previously characterized locus *Sad3/TG1* (24). We sequenced four independent *sad3* mutants, mapped the sequencing reads to our genome assembly and called SNVs. An optional stringency filter of our pipeline allows filtering for GC to AT transitions, which are the major type of mutation expected from sodium-azide (38, 39). We applied this filter and used a 10-kb sliding window to find regions in the genome where all sequenced mutants had a variation with regard to the wild type. This resulted in a single interval. Alignment to *Sad3/TG1* (GenBank: QHG10988.1) (24) confirmed our interval to be the correct locus.

We then sequenced the genomes of the four *sad4-like* mutants that we had identified (#370, 681, 1212, 1467) and analyzed the data following the same pipeline described for *Sad3/TG1* (24). This resulted again in a single candidate gene (*AS07G071450*) which was predicted to encode a phenylalanine ammonia lyase (PAL). The locus was further confirmed by cloning and sequencing (Table 1). All four amino acid changes were predicted using the SIFT algorithm (33) to affect protein function.

**Fig. 4.**
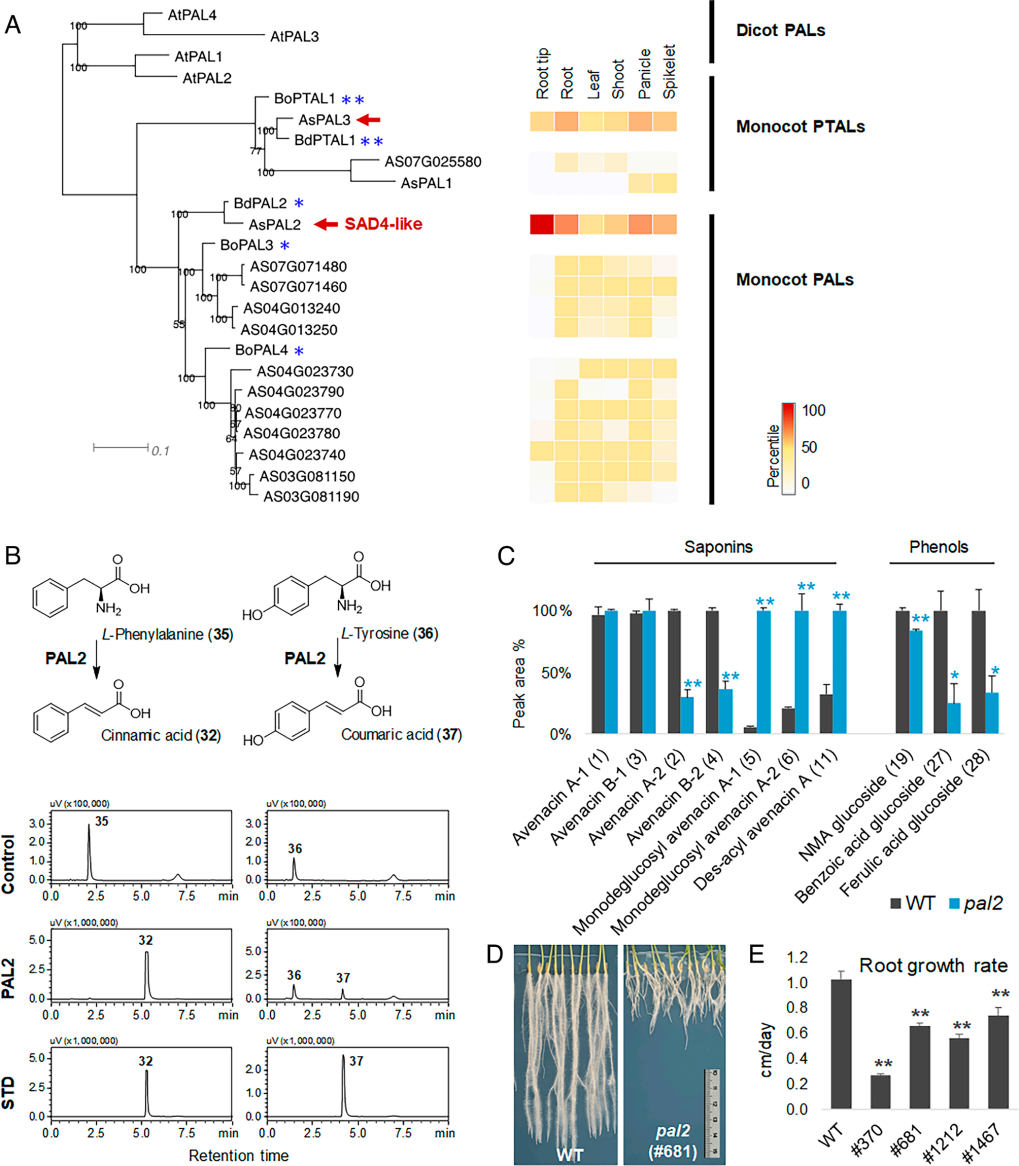
Mutant chemotype, expression pattern, phylogenetic analysis, and functional analysis for *Pal2*. (*A*) Phylogenetic analysis of 15 predicted *A. strigosa* PAL genes identified by transcriptome mining (*Left*) and their expression patterns (*Right*). **, reported bifunctional phenylalanine/tyrosine ammonia-lyase (PTAL); *, reported monofunctional phenylalanine ammonia-lyase (PAL); Red arrows indicate enzymes characterized in this study. For accession numbers, *SI Appendix,* Table S6. (*B*) In vitro functional analysis of PAL2. Control, reactions with the addition of boiled PAL2. STD, reference standards. (*C*) Relative content of avenacins and phenols in root extracts of the wild type and *pal2* mutants. Peak areas (±SE) are from untargeted metabolomics analysis (for WT, *n* = 3 biological replications; for *pal2* mutant, *n* = 3 different lines #681, #1212, #1467). *P < 0.05, **P < 0.01 comparing to the wild type (Student’s *t* test). (*D*) Roots of 12-d-old oats growing on water agar plates. (*E*) Growth rates of the roots.

PAL enzymes serve as the entry point for the phenylpropanoid pathway (40, 41). A tBLASTn search of the *A. strigosa* transcriptome data using the candidate *SAD4-like* gene as the query sequence yielded 14 additional sequences (*E* value = 0) (Fig. 4*A*). One of these (*AS06G006190*) corresponds to a previously reported PAL enzyme from hexaploid oat, *AsPAL1* (42). The *SAD4-like* gene was therefore named *PAL2*. *PAL2* is expressed across all six tissues examined, with highest expression in the root tips. A third gene *PAL3* (*AS07G071360*) is also expressed at lower levels across the six tissues, while the expression levels of the other genes (including *AsPAL1*) are relatively low. PAL2 was cloned, expressed in *Escherichia coli*, and shown to catalyze effective conversion of *L*-phenylalanine to cinnamic acid (Fig. 4*B*). It had only limited activity when presented with *L*-tyrosine as a potential substrate. The product peak area ratio for *L*-phenylalanine compared to *L*-tyrosine was 67.6, indicating that PAL2 is a monofunctional ammonia lyase with a preference for *L*-phenylalanine. In contrast, PAL3 had activity toward both *L*-phenylalanine and *L*-tyrosine (*SI Appendix,* Fig. S15) suggesting that it is a bifunctional PAL. This was consistent with phylogenetic analysis, which showed that PAL2 and PAL3 cluster with mono- and bifunctional PALs from *Brachypodium distachyon* (43), respectively (Fig. 4*A* and *SI Appendix,* Table S6). Alignment of the PAL2 and PAL3 amino acid sequences with those of other characterized PAL and PTAL enzymes showed that the key residues associated with substrate specificity were consistent with previous findings (41) (*SI Appendix,* Fig. S16). The expression of PAL2 in *N. benthamiana* boosted the production of benzoic acid *O*-glucoside (**27**), cinnamic acid *O*-glucoside (**31**), and coumaric acid *O*-glucoside (**38**), all of which were synthesized in the phenylpropanoid pathway downstream of PAL2 (*SI Appendix,* Fig. S13).

Root extracts of *sad4-like/pal2* mutants have reduced levels of phenolic acids when compared to the wild type. The impact on benzoic acid glucoside and ferulic acid glucoside is much more pronounced (>65% decrease) than for *N*-methyl anthranilic acid glucoside (17% decrease, Fig. 4*C*). This is consistent with our finding that the levels of avenacins A-2 and B-2, but not A-1 and B-1, are reduced in *sad4-like/pal2* mutants. Collectively our results suggest that PAL2 functions in the biosynthesis of phenolics, specifically in the phenylpropanoid pathway for benzoic acid synthesis, rather than in the tryptophan pathway for *N*-methyl anthranilic acid synthesis (28).

We previously reported that mutants affected at *TG1/Sad3* and *Sad4* (but not the other avenacin pathway genes) have stunted roots, the effects being less severe for *sad4* mutants compared to *sad3* mutants (18). All four *pal2* mutants (#370, 681, 1212, 1467) also had substantially reduced root growth (Fig. 4 *D* and *E*). We also investigated the lignin content of the wild type and mutant lines. TILLING mutants of hexaploid oat with SNVs in *AsPal1* have previously been shown to have reduced lignin content in the seeds, although other plant tissues were not examined (42). The lignin content of the *pal2* mutants in this study appeared somewhat lower for the roots, but overall there was no significant reduction for either roots or leaves (*SI Appendix,* Fig. S17). Of note, lignin biosynthesis via PAL is likely to be regulated by multiple factors, including metabolic feedback (40, 43). In contrast, the effects of *Pal2* mutation on benzoic *O*-glycoside (**27**) and ferulic acid glycoside (**28**) were clear (Fig. 4*C*). Thus mutation of *pal2* affects phenolic acids—key building blocks of avenacin—more markedly that it impacts total lignin, providing further support for a more specialized auxiliary role for PAL2 in avenacin biosynthesis.

## Discussion

The core avenacin pathway is encoded by a 12-gene BGC in oats and is required for disease resistance. It has evolved since the divergence of oats from other cereals and grasses (15). Here, we clone and characterize two nonclustered genes involved in avenacin biosynthesis, *Sad4* and *Pal2*, which encode a broad-substrate UGT84 family glucosyltransferase and a monofunctional phenylalanine ammonia lyase, respectively. Our evidence indicates that these two enzymes contribute to avenacin biosynthesis by synthesizing the acyl sugar donors required for avenacin glycosylation and acylation. The 12 genes in the avenacin BGC are expressed specifically in the epidermal cells of the root tips and the lateral root initials, and are located at the tip of the long arm of chromosome 1 of *A. strigosa* (15). In contrast, *Sad4* and *Pal2* are expressed in multiple tissues including the roots, root tips, leaves, shoots, panicles, and spikelet, and are located on chromosomes 4 and 7, respectively (15).

Glucosides of benzoic acid (**29**) and *N*-methyl anthranilic acid (**20**) are the presumed acyl donors for SAD7, the serine carboxypeptidase-like acyltransferase that adds the acyl groups to the triterpene scaffold (19). The sugar donor for the noncanonical sugar transferase SAD3, a transglucosidase, is also expected to be an acyl sugar (24). The decrease in acyl sugars (*N*-methyl anthranilic acid *O*-glucoside (**19**), benzoic acid *O*-glucoside (**27**), and ferulic acid *O*-glucoside (**28**) and concomitant accumulation in avenacin pathway intermediates [de-acyl avenacin A (**11**) and monodeglucosyl avenacin A-1/A-2 (**5**/**6**)] in root extracts from *sad4* and *pal2* mutant lines is consistent with this scenario.

**Fig. 5.**
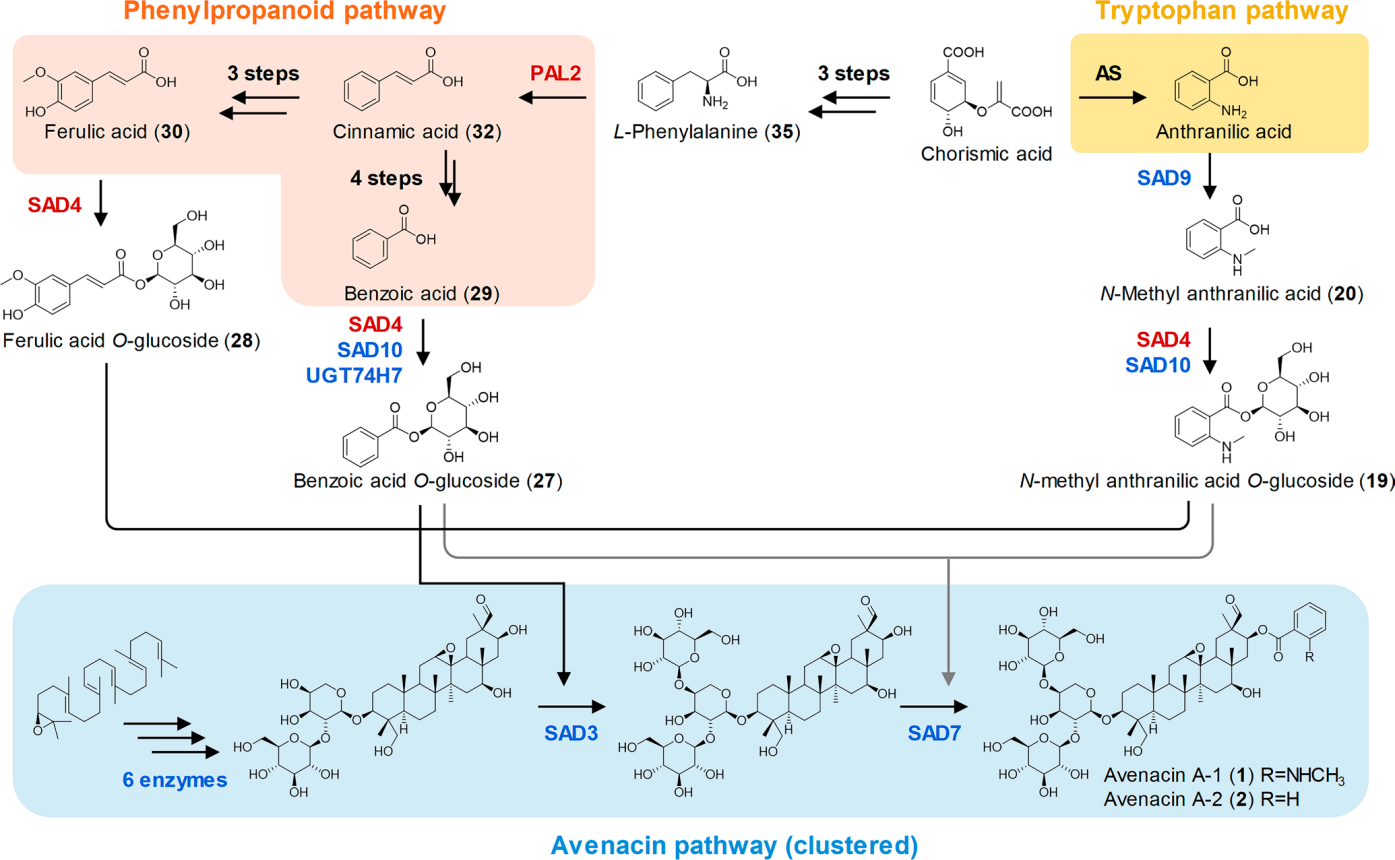
The roles of the CAEs SAD4 and PAL2 in avenacin biosynthesis. See (28) for a review of the phenylpropanoid and tryptophan pathways, and (15) for the full avenacin pathway elucidation. AS, anthranilate synthase.

PAL2 is the first committed enzyme in the phenylpropanoid pathway, and its downstream products can give rise to a diverse array of phenolic scaffolds (28). The gene expression level of *PAL2* was consistently the highest among the six tissues we examined, compared to other oat *PAL* genes (Fig. 4*A*), highlighting the pivotal role of PAL2 in this pathway. SAD4, identified as one of the UGT84 family members with the broadest substrate spectrum, catalyzes the formation of glucose ester bonds using substrates derived from both the phenylpropanoid and tryptophan pathways (Fig. 3*C* and *SI Appendix,* Fig. S11). Heterologous expression of SAD4 and PAL2 in *N. benthamiana* also enhanced the production of acyl sugars such as benzoic acid *O*-glucoside (**27**) and coumaric acid *O*-glucoside (**38**), suggesting their in vivo functions likely align with their in vitro functions (*SI Appendix,* Fig. S13). PAL2 and SAD4 exhibit a broader expression spectrum in oats and influence a wider range of reactions. Because these enzymes do not form part of the core avenacin pathway but rather supply the precursors needed for avenacin acylation and glycosylation, we have termed them Cluster Auxiliary Enzymes (CAEs) (Fig. 5). Their involvement in avenacin biosynthesis reveals the intricate interplay between general plant metabolism and this BGC-encoded pathway. In pathogenicity tests with the wheat pathogen *Gaeumannomyces graminis* var. *graminis sad4* and *pal2* mutants showed increased susceptibility compared to the wild type *A. strigosa* line (*SI Appendix,* Fig. S18). They were not, however, as susceptible as *sad1* and *sad2* mutants, which completely lack avenacins (25).

To date, we have assigned a total of 85 mutants to characterized avenacin genes, including nine of the BGC genes and the *Sad4* and *Pal2* genes characterized here. Overall, we observe a linear correlation between gene length and the number of independent mutant alleles recovered (*R* = 0.76) (*SI Appendix,* Fig. S19). Mutants for the remaining three cluster genes have not as yet been identified, probably due to functional redundancy. For example, the two sugar transferases encoded by the avenacin BGC genes *UGT74H5* (*SAD10*) and *UGT74H7* are known to share overlapping functions (21). The genome and transcriptome resources that we have generated for *A. strigosa* accession S75 coupled with this comprehensive mutant collection now open up opportunities for further investigation of the function and regulation of this paradigm BGC.

## Materials and Methods

### Plant Material.

Wild type and mutant *A. strigosa* lines were grown as previously described (25). A complete list of mutant lines is included in *SI Appendix,* Table S1.

### DNA Analysis.

Genomic DNA was isolated from 6-d-old seedlings of *A. strigosa* using the DNeasy Plant Mini kit (Qiagen). The WT and mutant forms of the previously characterized avenacin biosynthetic genes *Sad1/bAS1*, *Sad2/ CYP51H10*, *Sad3*/*TG1*, *Sad4*, *Sad6/CYP72A475*, *Sad7/ SCPL1*, and *Sad9/MT1* (16, 17, 19, 20, 23, 24) were amplified by PCR. The PCR products were purified and sequenced using sets of sequencing primers. Each mutant gene was sequenced to at least twofold coverage. The primers used for amplification and sequencing can be found in *SI Appendix,* Table S7. All standard sequencing were performed by Eurofins Genomics.

### Gene Accession Numbers.

*Sad4* (Genbank EU496495.1, AS01G000200), *Pal2* (Genbank PP746582, AS07G071450), *Pal3* (Genbank PP746583, AS07G071360).

### Chemicals.

Standards of avenacin A (**1**), benzoic acid (**29**), ferulic acid (**30**), *N*-methyl anthranilic acid (**20**), cinnamic acid (**32**), *L*-phenylalanine (**35**), *L*-tyrosine (**36**), *p*-coumaric acid (**37**), scopoletin, anthranilic acid, *p*-hydroxybenzoic acid, luteolin, naringenin, biochanin A (5,7-dihydroxy 4′-methoxy isoflavone), and digitoxin were obtained from Sigma-Aldrich. Standards of apigenin (**34**) and caffeic acid were from TCI (Oxford, UK); kaempferol was from Fluka (Germany), isoquercitrin, emodin, and indole-3-butyric acid were from Must Bio-Technology (Chengdu, China); and apigeninidin was from Extrasynthese (Genay, France). Benzoic acid *O*-*β*-D-glucoside (**27**) and monodeglucosyl avenacin A-1 (**5**) have been purified previously (18, 21, 24). *N*-methyl anthranilic acid *O*-*β*-D-glucoside (**19**) and apigenin 7-*O*-*β*-D-glucoside (**33**) were purified from large-scale enzyme reactions as described in *SI Appendix Supplement* 1. Other chemicals and solvents were purchased from Sigma-Aldrich or Merck Millipore unless otherwise stated.

### Targeted LC/MS Analysis and Data Analysis.

Metabolites were extracted from 5-d-old oat seedlings. The roots of each line were freeze-dried and 1.0 mg aliquots were extracted in 400 µL of methanol for 1 h by ultrasonication. The extracts were then centrifuged (13,000 rpm, 5 min), and the supernatants dried under vacuum. The residues were reconstituted in 300 µL of water and extracted twice with 200 µL aliquots of hexane. The hexane layer was discarded. The aqueous layer was then dried down, reconstituted in 100 µL of methanol, and mixed with 100 µL of water. Aliquots of 10 µL were injected for LC/MS analysis.

Targeted LC/MS analysis was carried out on a Nexera X2 HPLC system connected to SPD-M20A UV detector and an LCMS-2020 single quadrupole mass spectrometer (Shimadzu). Oat root extracts were separated on a Kinetex XB-C_18_ analytical column (50 × 2.1 mm, 2.6 μm, Phenomenex). Acetonitrile (A) and water (B), each containing 0.1% formic acid, were used as mobile phases. The gradient elution program was 0 min, 20% A; 3 min, 20% A; 28 min, 60% A; 30 min, 100% A; 33 min, 100% A. The flow rate was 0.3 mL/min and the column oven was set at 30 °C. The mass spectrometer was operated in positive-negative switching mode scanning from *m*/*z* 50 to *m*/*z* 1,500. The postcolumn split ratio was 1:1. Peak areas of avenacin intermediates (*SI Appendix,* Fig. S1) were extracted using LabSolutions v5.72 (Shimadzu), and then used for two-component principal component analysis using SIMCA 14.1 demo (Umetrics).

### GC/MS Analysis.

Metabolites were extracted from 5-d-old oat seedlings and analyzed as described in (44). Aliquots (2.0 mg) of freeze-dried root material were extracted with 500 µL of saponification reagent (10% (*w*/*v*) KOH in 90% (*v*/*v*) ethanol), and incubated at 70 °C for 2 h. The metabolites were subsequently extracted with 250 µL of water and 500 µL of hexane. For GC/MS analysis, 150-μL aliquots of the hexane extract were dried, and the residues were resuspended in 100 μL of Tri-Sil Z reagent before incubating at 70 °C for 30 min. GC-MS analysis was performed on an Agilent GC (7890B)-MSD (5977A) equipped with a Zebron ZB5-HT Inferno column (35 m × 0.25 mm, 0.1 μm film thickness, Phenomenex). A 1-μL aliquot of each sample was injected into the GC inlet (250 °C) in pulsed splitless mode. The oven temperature program was as follows: 0 min at 170 °C; hold at 170 °C for 2 min; ramp to 300 °C at a rate of 20 °C/min; hold at 300 °C for another 11.5 min. The mass spectrometer scanned over a range of *m*/*z* 60-800.

### Untargeted LC/MS Analysis and Data Analysis.

Metabolites were extracted from 5-d-old oat seedlings. Aliquots of freeze-dried roots (2 mg) or freeze-dried shoots (3 mg) were extracted in 400 µL of methanol (containing 0.5 µg/mL of digitoxin as internal standard) for 1 h. Other extraction steps were the same as for “Targeted LC/MS analysis and data processing.”

LC/MS analyses were carried out on a Nexera X2 HPLC system coupled with an IT-TOF mass spectrometer (Shimadzu). Samples were separated using a gradient program: 0 min, 20% A; 3 min, 20% A; 28 min, 50% A; 30 min, 95% A; 34 min, 95% A. Other settings were the same as *Targeted LC/MS analysis and data analysis*. The mass spectrometer was operated in positive-negative switching mode scanning from *m*/*z* 200 to *m*/*z* 1500 for metabolomics analysis, and positive or negative MS/MS mode for compound identification and mass spectral networks analysis. Peak areas were extracted using Profiling Solution v1.1 (Shimadzu).

Mass spectral networks were analyzed by Global Natural Products Social Molecular Networking server (26) (GNPS, https://gnps.ucsd.edu/) using the following parameters: parent mass tolerance, 1 Da; fragment ion tolerance = 0.5 Da; minimum pair cosine, 0.6; minimum matched peaks, 3; minimum cluster size, 2; minimum peak intensity, 25. Network data were visualized and cropped in Cytoscape v3.5.1 (45) (http://www.cytoscape.org/) to select the avenacin-related cluster and to remove nodes with the same molecular weights (isomers).

### Analysis of Segregating Progeny.

F_2_ progeny (192 seeds) derived from a cross between the wild type *A. strigosa* accession S75 and the *sad4* mutant #933 were used for segregation analysis. For chemotyping, individual root tips from 5-d-old seedlings (~1 cm) were immersed in 100% methanol of 1 h. The UV fluorescent avenacins A-1 and B-2 were visualized under UV light at 365 nm. For genotyping, part of the *Sad4* gene was amplified from DNA extracts and genotyped for a characterized single nucleotide polymorphism by sequence analysis.

### Phylogenetic Analysis and RNA-Seq Analysis.

The RNA-seq data used were from our previous study (http://db.ncgr.ac.cn/oat/RNAseq) (22). Phenylalanine ammonia lyase amino acid sequences were retrieved by tBLASTn using *PAL2* as the template and the RNA-seq as the database. The phylogenetic tree shown in Fig. 3*C* was constructed using the neighbor-joining method and the Jukes–Cantor protein distance measure method in CLC Main Workbench v7.9.1 (Qiagen). Bootstrap analysis was performed with 100 bootstrap replicates. For the phylogenetic tree shown in Fig. 4*A*, alignments were done using Muscle with the option -maxiters 100 (46). RaXML was used for the trees, using the PROTGAMMAAUTO model and 100 rapid bootstraps (47).

### Cloning, Expression, and Protein Purification.

Total RNA was extracted using the Trizol and RNAeasy Kit (Qiagen) from roots of 5-d-old oat seedlings according to the manufacturer’s instructions. The extract was treated with DNase (Qiagen). cDNA libraries were prepared using an Affinityscript QPCR cDNA synthesis kit (Agilent Technologies) according to the manufacturer’s instructions. The coding regions of *Sad4, Sad10, UGT74H7*, *PAL2,* and *PAL3* were amplified from total cDNA using Gateway primers (*SI Appendix,* Table S7) and cloned into pDONR207 vectors using BP clonase II enzyme mix (Invitrogen) according to the manufacturer’s instructions. After verification of the sequences, the genes were cloned from pDONR207 into pH9-GW vectors using the LR clonase II enzyme (Invitrogen) according to the manufacturer’s instructions. The plasmids were then transformed into *E. coli* BL21 strains (Novagen): BL21 for *Sad10*, Rosetta 2(DE3) for *Sad4* and *UGT74H7*; Rosetta 2(DE3) pLysS for *PAL2* and *PAL3*. The strains were grown at 37 °C, 200 rpm in LB-media containing the corresponding antibiotics (50 μg/mL kanamycin for BL21, 50 μg/mL kanamycin, and 35 μg/mL chloramphenicol for Rosetta) until they reached an OD of 0.4 to 0.6. For the glucosyltransferases, expression of the recombinant proteins was induced by the addition of 0.5 mM isopropyl β-D-thiogalactoside (IPTG), and then the cells were incubated for 8-16 h at 16 °C. For the phenylalanine ammonia-lyases, protein expression was induced by the addition of 0.5 mM IPTG, and the cells were incubated for 6 h at 28 °C. Following expression, the cells were harvested by centrifugation at 3,220×*g* for 15 min at 4 °C.

For protein purification, cells from 100 mL culture were resuspended in 6 mL of sonication buffer (pH 7.8). The sonication buffer contained 300 mM NaCl, 50 mM Tris buffer, 20 mM imidazole, 5% glycerol, 50 µL of Tween 20, and one tablet of EDTA-free protease inhibitor (Roche). Cells were disrupted by sonication in an ice bath and the debris was removed by centrifugation at 12,000×*g* and 4 °C for 20 min. The soluble fraction was mixed with 150 µL of nickel-charged resin (Ni-NTA agarose, Qiagen) preequilibrated with the sonication buffer, and incubated at 4 °C for 1 h. The resin was then washed with 500 µL of buffer A (300 mM NaCl, 50 mM Tris buffer, 20 mM imidazole, 5% glycerol, pH 7.8) three times, and the protein eluted with 100 µL buffer B (300 mM NaCl, 50 mM Tris buffer, 500 mM imidazole, 5% glycerol, pH 8.0).

### Glucosyltransferase Activity Assays.

To investigate the substrate specificities of SAD4, SAD10, and UGT74H7, assays were carried out in uniform conditions. The assays contained Tris-HCl (50 mM, pH 6.5), UDP-glucose (500 μM), MgCl_2_ (5 mM), substrate (200 μM), and recombinant GT protein (0.056 ng/µL) in a total volume of 50 μL. The mixtures were incubated at 37 °C for 1 h and reactions were then terminated by adding 200 μL of chilled methanol. The assay mixtures were dried down under vacuum and reconstituted in 100 μL of 10% acetonitrile. Aliquots (10 μL) were analyzed by LC/UV/MS. The instrument methods and mobile phases are described in *Targeted LC/MS analysis and data analysis*. For substrate screening, samples were separated on a Kinetex XB-C_18_ analytical column (50 × 2.1 mm, 2.6 μm) with an elution program of 0 min, 8% A; 14 min, 25% A; 14.5 min, 50% A; 17.5 min, 50% A; flow rate, 0.3 min/mL. Chromatograms were recorded by HPLC/UV at 270 nm for **29**, **32,** and **34**, by HPLC/fluorescence detector (Shimadzu) at Ex 353 nm, Em 441 nm for **20**, and by LC/MS for other compounds. For reaction condition optimization and kinetic studies, see *SI Appendix Supplement* 2 for details.

### Phenylalanine Ammonia-Lyases Activity Assays.

Reactions contained Tris-HCl (50 mM, pH 8.5), substrate (5 mM), and equal concentrations of the PAL2 and PAL3 proteins (48 ng/µL) in a total volume of 100 μL. Assays were incubated at 50 °C for 2.5 h and the reactions terminated by addition of 400 μL of chilled methanol. They were then dried under vacuum and reconstituted in 100 μL of water. Aliquots (10 μL) were analyzed by LC/UV/MS. All instrument methods were as described in *Targeted LC/MS analysis and data analysis*. The Kinetex EVO C_18_ analytical column (100 × 2.1 mm, 2.6 μm) was used with an elution program of 0 min, 2% A; 5 min, 55% A; 5.5 min, 2% A; 8 min, 2% A. Cinnamic acid, coumaric acid, and caffeic acid were detected at 260 nm, 290 nm, and 320 nm, respectively.

### Heterologous Expression in *N. benthamiana*.

*Sad4* and *Pal2* were cloned from pDONR207 into pEAQ-*HT*-DEST1 vectors. The expression constructs were transformed into *Agrobacterium tumefaciens* strain LBA4404 by flash-freezing in liquid nitrogen. Strains carrying the relevant constructs were cultured and prepared for agro-infiltration as described previously (44).

Leaves were harvested 5 d after agro-infiltration and freeze-dried. Aliquots of dried leaf material (10 mg) were weighed and extracted with 1 mL of methanol by sonication for 1 h. The supernatants were dried under vacuum, and the residues reconstituted in 70 µL of methanol, and mixed with another 70 µL of water. The extracts were analyzed by LC/IT-TOF-MS (Shimadzu). Samples were separated on a Kinetex EVO C_18_ analytical column (100 × 2.1 mm, 2.6 μm) with an elution program of 0 min, 3% A; 1 min, 3% A; 20 min, 20% A. Flow rate, 0.3 mL/min. For details of other settings see “*Untargeted LC/MS analysis and data analysis*”.

### Mutational Genomics to Identify PAL2.

Sequencing was performed on an Illumina platform with 150 bp paired-end reads. An average of 383 and 168 Gb data was generated per mutant for *Sad3* and *Sad4-like*, respectively, equivalent to 95- or 42-fold coverage. The raw data were deposited in the European Nucleotide Archive under study number (PRJEB45039). The mutational genomics analyses are described in *SI Appendix Supplement* 3. The scripts are deposited on GitHub (https://github.com/steuernb/oat_mutseq/).

### Lignin Quantification.

Lignin quantification was carried out as previously described (48) with slight modifications. In brief, roots and leaves of 28-d-old seedlings were freeze-dried overnight and then bead milled with tungsten carbide beads at 1,000 rpm for 10 min (Horiba). Aliquots (1 mg) of dried tissue powder were transferred to 1.5 mL microcentrifuge tubes, were washed with 1 mL of 80% ethanol in a shaking dry bath (1,200 rpm, 15 min), centrifuged (10,000*×g,* 15 min), and the supernatant decanted. The ethanol extraction step was repeated four times, followed by a single wash with chloroform/methanol (2:1, 1 mL) before rinsing with acetone (0.5 mL). Pellets were air dried at 42 °C, suspended in 1 mL of 10 mM PBS (NaCl, 8.1 g/L; KCl, 0.2 g/L; phosphate buffer, 11.76 mM; pH 7.4) and heated in a dry bath (95 °C, 30 min) to gelatinize starch. Samples were cooled to 55 °C and treated with α-amylase (1 µL, 14.7 U; Sigma-Aldrich A3403), amyloglucosidase (20 U; Sigma-Aldrich 10115; 70 U mg^−1^), and pullulanase (10 µL, 18 U; Sigma-Aldrich E2412) for 2 h. They were then heated to 100 °C for 10 min, centrifuged at 14,000×*g*, and the supernatant discarded. The pellet was washed three times with dH_2_O, rinsed with 200 µL of acetone and dried at 42 °C in a dry bath. 1 mg aliquots were transferred to 2 mL screw-cap microcentrifuge tubes and 100 µL of acetyl bromide solution (25% v/v in glacial acetic acid) added before incubation in an oven at 50 °C for 2 h. The tubes were then transferred to a shaking dry bath (300 rpm, 50 °C) for a further hour. After cooling to room temperature on ice, 400 µL of 2 M sodium hydroxide and 70 µL of freshly prepared 0.5 M hydroxylamine hydrochloride was added to each tube, the tubes were vortexed, and then 1.43 mL of glacial acetic acid was added. Following further vortexing and centrifugation at 10,000×*g* for 10 min, 200 µL of the solution was transferred to a 96-well plate and absorbance at 280 nm measured immediately using a ClarioStar microplate reader (BMG Labtech).

### Pathogenicity Assays.

Pathogenicity assays were carried out as described in (49). The fungal strain used for the pathogenicity assay was *G. graminis* var. *tritici* T1-1 (50). The fungus was cultured on potato dextrose agar at 22 °C for 5 d, and mycelial plugs were taken from the actively growing colony margin with a sterile No. 3 cork borer (7.5 mm diameter). Seeds were surface-sterilized by immersion in ethanol for 1 min, washing with sterile water three times, then soaking in bleach (5% hypochlorite) for 5 min before washing three more times with sterile water. Plastic centrifuge tubes (50 mL) were filled with 30 mL of sterile vermiculite. Two mycelial plugs were placed on top and covered with a further 5 mL of vermiculite. Two surface-sterilized seeds were placed on top of the vermiculite, followed by a further 5 mL of vermiculite to cover and the tubes watered with 10 mL of sterile water. A total of five replicate tubes were set up for each oat line. Tubes were covered with Parafilm and incubated at 22 °C in a controlled growth chamber (photoperiod of 16 h light, 8 h dark, 70 % relative humidity) for 4 wk. Seedling roots were then washed in water and examined for lesions. Pathogenicity was scored on an arbitrary scale of 0 to 8 as previously described (51) where; 0, no disease symptoms; 1, some root browning; 2, a single lesion visible on the roots; 3, multiple lesions visible; 4, multiple lesions, with one lesion confluent with the seed; 5, multiple lesions, with more than one lesion confluent with the seed; 6, extensive root necrosis and some browning of leaf sheath; 7, extensive root necrosis, leaves wilting and chlorotic; 8, extensive necrosis of roots and leaves.

## Supplementary Material

Appendix 01 (PDF)

## Data Availability

All study data are included in the article and/or *SI Appendix*.
