# Supplementary material for: Comprehensive mutant chemotyping reveals embedding of a lineage-specific biosynthetic gene cluster in wider plant metabolism
Source: Proc Natl Acad Sci U S A. 2025 Mar 19;122(12):e2417588122. doi: 10.1073/pnas.2417588122 (PMC11962460; doi:10.1073/pnas.2417588122)
Supplement: Supplementary file 1 — Appendix 01 (PDF) [file pnas.2417588122.sapp.pdf]

PNAS

Supporting Information for:

Comprehensive mutant chemotyping reveals embedding of a lineage-specific biosynthetic gene cluster in wider plant metabolism

Xue Qiao[a,b,*], Alan Houghton[a], James Reed[a], Burkhard Steuernagel[c], Jiahe Zhang[b], Charlotte Owen[a], Aymeric Leveau[a], Anastasia Orme[a,], Thomas Louveau[a], Rachel Melton[a], Brande B. H. Wulff [d], and Anne Osbourn[a,*]

*Co-corresponding authors. **Email:** qiaoxue@bjmu.edu.cn; anne.osbourn@jic.ac.uk

This PDF file includes:

Supporting text
Figures S1 to S26
Tables S1 to S7

Supporting Information Text

Supplement 1

Purification of SAD4 products and structural characterization

Apigenin 7-*O*-*β*-D-glucoside (~5.0 mg) were produced by 80-mL scaled up enzyme reactions. The products were purified by reverse-phase semi-preparative HPLC. Apigenin 7-*O*-*β*-D-glucoside (HR-MS *m*/*z* 431.0983, calcd for $C_{21}H_{19}O_{10}^-$, [M-H]$^-$) was characterized by NMR (*SI Appendix* Fig. S21-25).

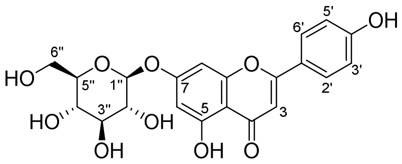

Apigenin 7-*O*-*β*-D-glucoside (~5.0 mg) were dissolved in DMSO-*d*$_6$ and analysed by NMR. ^{1}H, ^{13}C and HMBC NMR spectra acquired on a Bruker 400 MHz Topspin NMR spectrometer. Apigenin 7-*O*-*β*-D-glucoside: ^{1}H-NMR (400 MHz, DMSO-*d*$_6$): 7.96 (2H, d, *J* = 9 Hz, H-2', H-6'), 6.96 (2H, d, *J* = 9 Hz, H-3', H-5'), 6.86 (1H, s, H-3), 6.83 (1H, d, *J* = 2 Hz, H-8), 6.44 (1H, d, *J* = 2 Hz, H-6), 5.06 (1H, d, *J* = 7 Hz, Glc-1''), 3.71 (1H, d, *J* = 9.6 Hz, Glc-6''b), 3.1-3.51 (5H, m, Glc-2'', Glc-3'', Glc-4'', Glc-5'', Glc-6''a). ^{13}C-NMR (100 MHz, DMSO-*d*$_6$): 182.0 (s, C-4), 166.6 (s, C-2),164.4 (s, C-5), 163.0 (s, C-7), 161.9 (s, C-4'), 157.0 (s, C-9), 128.6 (2d, C-2', C-6'), 121.6 (s, C-1'), 116.1 (2d, C-3', C-5'), 105.4 (s, C-10), 103.0 (d, C-3), 99.9 (d, Glc-1''), 99.5 (d, C-6), 94.8 (d, C-8), 77.2 (d, Glc-5''), 76.4 (d, Glc-3''), 73.1 (d, Glc-2''), 69.6 (d, Glc-4''), 60.6 (t, Glc-6''). HMBC H-2',6'/C-4',6'/2'; H-3',5'/C-1', 5'/3', 2; H-3/C-2, 4, 10, 1'; H-8/C-9; H-6/C-5, 8, 10, H-1''/C-7. Negative ion HR-MS *m*/*z* 431.0983 (calcd for $C_{21}H_{19}O_{10}^-$ [M-H]$^-$, *m*/*z* 431.0984).

N-methyl anthranilic acid *O*-*β*-D-glucoside (~2.5 mg) was produced by 80-mL scaled up enzyme reactions. The products were purified by reverse-phase semi-preparative HPLC. The identity of *N*-methyl anthranilic acid *O*-*β*-D-glucoside (HR-MS *m*/*z* 336.1054, calcd for $C_{14}H_{19}NO_7Na^+$, [M+Na]$^+$) was confirmed by analysing the mass data (20) (*SI Appendix* Fig. S26).

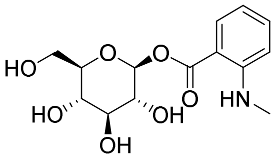

Supplement 2

Determination of kinetic parameters for SAD4

Purification of SAD4 for kinetic studies was carried out by liquid chromatography (ÄKTA purifier system, GE Healthcare) using an ion exchange column (1 mL HiTrap Chelating HP, GE Healthcare). The soluble fraction from the cell lysate was passed through a 0.2 μm syringe filter (Millipore). The fraction was then loaded onto a precooled column charged with Ni^{2+} solution, and equilibrated with buffer A as described above. A linear gradient of imidazole was used for elution by mixing buffer A and buffer B. Gradient program: 0 min, 0% buffer B; 8 min, 0% buffer B; 22.5 min, 60% buffer B; 23-25 min, 100% buffer B. The flow rate was 0.5 mL/min and the elution was monitored with a UV detector at 280 nm. Fractions containing SAD4 protein were collected and concentrated using 10000 DWA cutoff membrane (Amicon Ultra-4 Centrifugal Filter Unit, Millipore). Protein purity was assessed by SDS-PAGE gel (Invitrogen). Protein concentrations were determined using the Bradford method (Bio-Rad). Purified protein was flash-frozen in liquid nitrogen and stored at -80°C.

To optimize the reaction conditions for SAD4, effects of pH, temperature and metal ions were studied using *N*-methyl anthranilic acid (200 μM) as a substrate in 50 μL reaction volumes. Reactions were carried out at pH 3.5, 4.5 (citric acid-sodium citrate buffer, 50 mM), 5.5, 6.0, 6.5, 7.0, 7.5, 8.0, 8.5 (Tris-HCl buffer, 50 mM) and 9.5 (Tris-NaOH buffer, 50 mM). To find the optimal temperature, the mixtures were incubated at different temperature ranges from 20-65°C and at 4°C. To test the effects of different metal ions, NaCl, $MgCl_2$, $CaCl_2$, and EDTA (5 mM) were added. Reactions were terminated by heating at 95°C for 30 min. Following addition of 100 μL water, 10 μL aliquots were injected for HPLC analysis. All experiments were performed in triplicate. *N*-methyl anthranilic acid and its glucoside were detected using a fluorescent detector (FLD, RF-20A module, Shimadzu). The excitation and emission wavelengths were set at 353 nm and 441 nm, respectively. Other instrument methods were as described in “Glucosyltransferase activity assays”. The Kinetex EVO C_{18} analytical column (100 × 2.1 mm, 2.6 u) was used, with an elution program of 0 min, 2% A; 5 min, 55% A; 5.5 min, 2% A; 8 min, 2% A. The optimized assay contained Tris-HCl (50 mM, pH 6.5), UDP-glucose (500 μM), $MgCl_2$ (5 mM), substrate (200 μM), and recombinant GT protein (0.056 ng/μL) in a total volume of 50 μL for substrate screening reactions (*SI Appendix* Fig. S20).

For kinetic studies of SAD4, each 50 μL reaction mix contained Tris-HCl (50 mM, pH 6.5), UDP-glucose (500 μM), $MgCl_2$ (5 mM), purified SAD4 protein (0.37 ng for *N*-methyl anthranilic acid, 1.48 ng for benzoic acid and cinnamic acid, 1.30 ng for apigenin), and varying substrate concentrations. The mixtures were incubated at 30°C for 15 min. The concentrations were 1.25-15 μM for *N*-methyl anthranilic acid, 10-100 μM for benzoic acid and cinnamic, and 8-32 μM for apigenin due to its poor solubility. The reactions were terminated by addition of 1 μL of 50% phosphoric acid, with the exception of the *N*-methyl anthranilic acid reactions, which were terminated as described in the last paragraph. Supernatants were analysed by HPLC/UV (270 nm for benzoic acid, cinnamic acid and apigenin) or HPLC/FLD (Ex353 nm, Ex441 nm for *N*-methyl anthranilic acid). All experiments were performed in triplicate. The kinetic parameters (Figure 3D) were calculated with the method of Lineweaver-Burk plot.

Supplement 3

Mutational genomics analyses

Paired-end reads from individual mutants were processed using bwa (https://github.com/lh3/bwa, version 0.7.12) and samtools (http://www.htslib.org/, version 1.7) with the following program calls:

```
bwa index reference.fasta
bwa mem reference.fasta read1.fastq read2.fastq > mapping.sam
samtools view -Shub -o mapping.bam mapping.sam
samtools sort -o mapping.sorted.bam mapping.bam
samtools rmdup mapping.sorted.bam mapping.rmdup.bam
samtools index mapping.rmdup.bam
samtools mpileup -f reference.fasta -BQ0 mapping.rmdup.bam > mutant.mpileup
```

Variations were called from the resulting "mpileup" files using a light-weight custom SNP-caller extracting all positions where coverage was at least 10 reads and the frequency of the reference allele was smaller than 70%.

We implemented a MutSeq program that processes input from our SNP caller, and uses a sliding-window approach to compute intervals containing variations in more than one mutant. Although we identified candidates for *SAD3* and *PAL2* using the highest stringency, the program allows to define stringency for filters. In the subsequent description of filters, the values in brackets are those used for identifying our genes:

Window size (10,000): the size of interrogated intervals.
Number of mutants (4): number of mutants that need a mutation for an interval to be reported.
Number of canonical mutations (4): number of mutants that need a single nucleotide variant (SNV) typical of sodium-azide and EMS (*i.e.* GC>TA).
Maximum reference allele frequency (0.01): maximum frequency of reference allele.
Number of mutants that may share a position (1): Since the probability of independent mutants having a mutation at the same position is very low, this is a useful filter to remove noise.

Coverage (*Sad3*: 50; *Pal2*:20): the sequencing coverage at a position to consider it as a SNV. Note, this is the second time coverage is selected for. While the very large "mpileup" files are filtered for coverage with less stringency, this filter here allows for rapid iterations of the pipeline on the smaller pre-filtered SNV data set and testing different parameter settings. Starting with an unreasonable stringency of 100 (*Sad3*) and 50 (*Pal2*), relaxing the coverage threshold to 50 and 20 led to the single candidates for *Sad3* and *Pal2*.
The scripts are deposited on GitHub (https://github.com/steuernb/oat_mutseq/).

Supplement 4

Additional analytical methods for SAD4 substrate screening

The reaction mixtures were analysed by a HPLC system (Agilent 1260). Samples were separated on a YMC-Pack ODS-A column (4.6 mm × 250 mm, 5 μm). The mobile phase consisted of methanol (A) and water containing 0.1% formic acid (*v*/*v*, B). The mobile phase gradient was as follows: 0-8 min, 20%-60% A; 8-15 min, 60 %-100%; 15-17 min, 100% A; 17-17.1 min, 100%-20% A; 17.1-20 min, 20% A. The flow rate was 1.0 mL/min. The column temperature was 35°C. The detection wavelength was 275 nm.

The mass spectra were collected by a Q-Exactive quadrupole-orbitrap mass spectrometer equipped with a heated electrospray ionization source (Thermo Fisher Scientific, USA). MS analysis was performed in negative ion mode with the following parameters: spray voltage: -3.5 kV; capillary temperature: 350°C; sheath gas: 45 arb; aux gas: 10 arb; probe heater temperature: 400°C; S-lens RF level: 60 V; resolution: 70000 for full MS and 17500 for MS/MS; scan range: *m*/*z* 100-1200; stepped NCE: 35 eV. Data were processed using Xcalibur™ 4.1 software (Thermo Fisher Scientific).

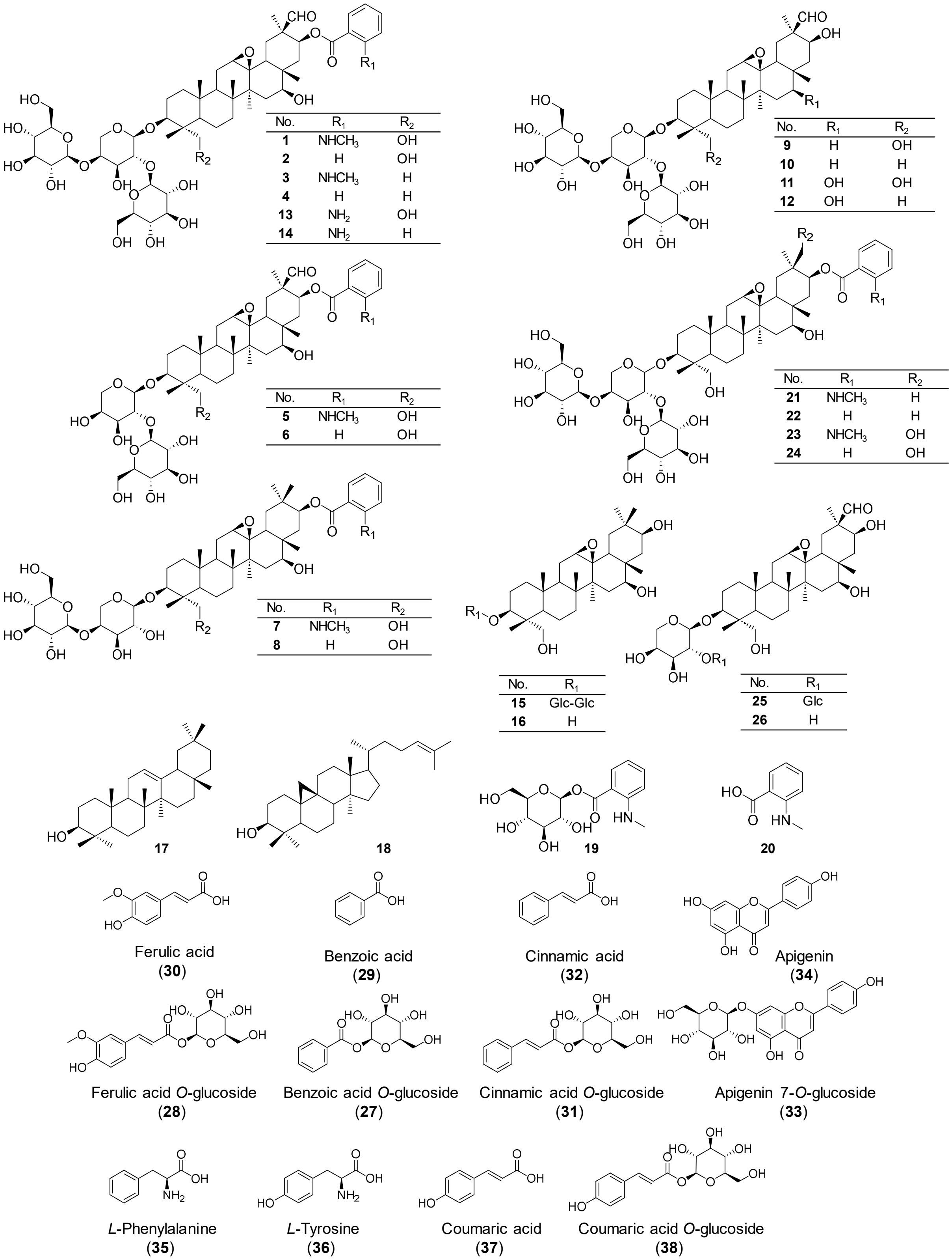

No.	R_1	R_2
**1**	$NHCH_3$	OH
**2**	H	OH
**3**	$NHCH_3$	H
**4**	H	H
**13**	NH_2	OH
**14**	NH_2	H

No.	R_1	R_2
**9**	H	OH
**10**	H	H
**11**	OH	OH
**12**	OH	H

No.	R_1	R_2
**5**	$NHCH_3$	OH
**6**	H	OH

No.	R_1	R_2
**21**	$NHCH_3$	H
**22**	H	H
**23**	$NHCH_3$	OH
**24**	H	OH

No.	R_1	R_2
**7**	$NHCH_3$	OH
**8**	H	OH

No.	R_1
**15**	Glc-Glc
**16**	H

No.	R_1
**25**	Glc
**26**	H

Fig. S1. Structures of avenacin intermediates (**1-26**) and other compounds (**27-38**) involved in this study.

No.	Name	Formula	MW	Accumulated in	Ref
**1**	Avenacin A-1	$C_{55}H_{83}NO_{21}$	1093.5458	WT	
**2**	Avenacin A-2	$C_{54}H_{80}O_{21}$	1064.5192	WT	
**3**	Avenacin B-1	$C_{55}H_{83}NO_{20}$	1077.5508	*cyp94d65*	
**4**	Avenacin B-2	$C_{54}H_{80}O_{20}$	1048.5243	*cyp94d65*	
**5**	Monodeglucosyl avenacin A-1	$C_{49}H_{73}NO_{16}$	931.4929	*sad3*	(24)
**6**	Monodeglucosyl avenacin A-2	$C_{48}H_{70}O_{16}$	902.4664	*sad3*	(24)
**7**	C30-H Monodeglucosyl avenacin A-1	$C_{49}H_{75}NO_{15}$	917.5137	*ugt91g6*	(24)
**8**	C30-H Monodeglucosyl avenacin A-2	$C_{48}H_{72}O_{15}$	888.4871	*ugt91g6*	(24)
**9**	Des-acyl, des-21-hydroxy avenacin A	$C_{47}H_{76}O_{19}$	944.4981	*sad6*	(23)
**10**	Des-acyl, des-21-hydroxy avenacin B	$C_{47}H_{76}O_{18}$	928.5032	*sad6*	(23)
**11**	Des-acyl avenacin A	$C_{47}H_{76}O_{20}$	960.4930	*sad7*	(19)
**12**	Des-acyl avenacin B	$C_{47}H_{76}O_{19}$	944.4981	*sad7*	(19)
**13**	Des-methyl avenacin A-1	$C_{54}H_{81}NO_{21}$	1079.5301	*sad9*	(20)
**14**	Des-methyl avenacin B-1	$C_{54}H_{81}NO_{20}$	1063.5352	*sad9*	(20)
**15**	C30-H Avenacin aglycone diglucoside	$C_{42}H_{70}O_{15}$	814.4715	*ugt99d1*	(22)
**16**	C30-H Avenacin aglycone	$C_{30}H_{50}O_{5}$	490.3658	*ugt99d1*	(22)
**17**	β-Amyrin	$C_{30}H_{50}O$	426.3862	*sad2*	(17)
**18**	Cycloartenol	$C_{30}H_{50}O$	426.3862	*sad1*	(16)
**19**	*N*-Methyl anthranilic acid *O*-glucoside	$C_{14}H_{19}NO_{7}$	313.1162	*sad7*	(19)
**20**	*N*-Methyl anthranilic acid	$C_{8}H_{9}NO_{2}$	151.0633	*sad7*	(19)
**21**	C30-H Avenacin A-1	$C_{55}H_{85}NO_{20}$	1079.5665	*cyp72a476*	proposed
**22**	C30-H Avenacin A-2	$C_{54}H_{82}O_{20}$	1050.5399	*cyp72a476*	proposed
**23**	C30-OH Avenacin A-1	$C_{55}H_{85}NO_{21}$	1095.5614	*cyp72a476*	proposed
**24**	C30-OH Avenacin A-2	$C_{54}H_{82}O_{21}$	1066.5349	*cyp72a476*	proposed
**25**	Des-acyl, monodeglucosyl avenacin A	$C_{41}H_{66}O_{15}$	798.4402	*sad3*	(this study)
**26**	Des-acyl, dideglucosyl avenacin A	$C_{35}H_{56}O_{10}$	636.3873	*sad7*	(this study)

Fig. S1 (continued). Structures of avenacin intermediates (**1**-**26**) and other compounds (**27**-**38**) in this study. ‘proposed’: intermediates proposed by analysing the substrates and products of the enzymes; ‘this study’: intermediates were identified by LC/MS in this study.

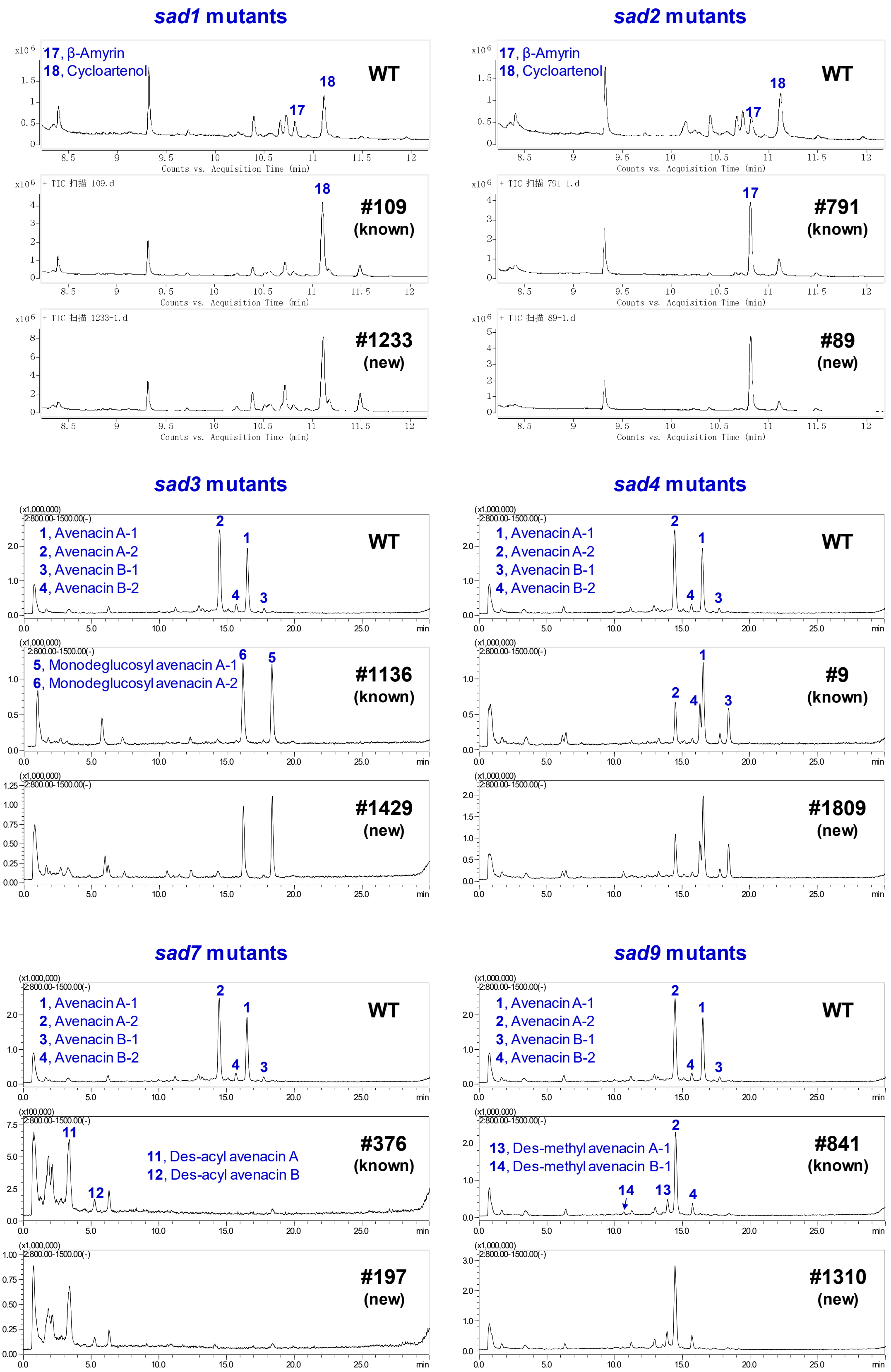

Fig. S2. Root extract chemical profiles for mutants identified in this study.

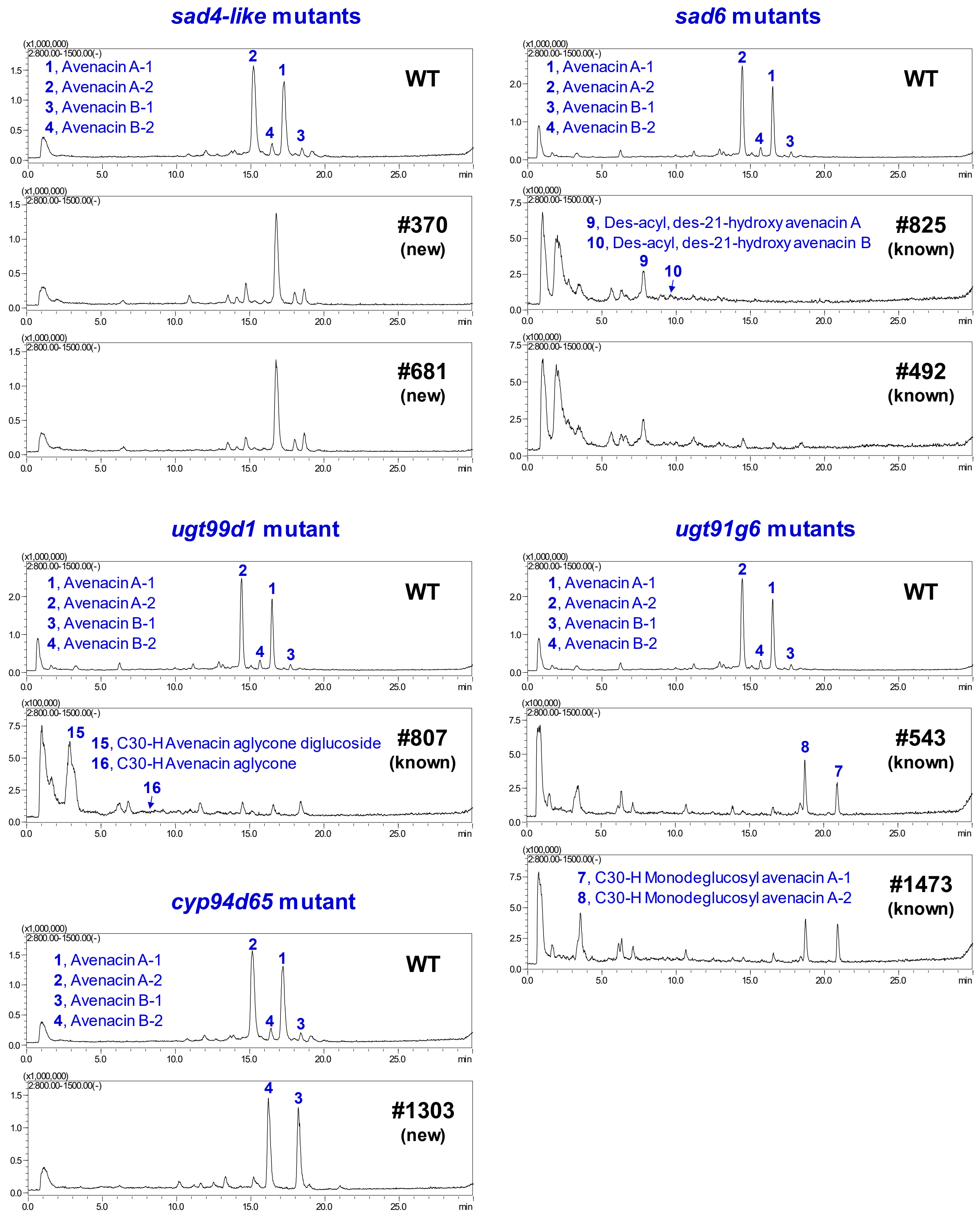

Fig. S2 (continued). Root extract chemical profiles for mutants identified in this study.

sad1 and *sad2* mutants were analysed by GC/MS (see 'GC/MS analysis' for details). The other mutants were analysed using LC/MS (see 'Targeted LC/MS analysis and data analysis' for details).

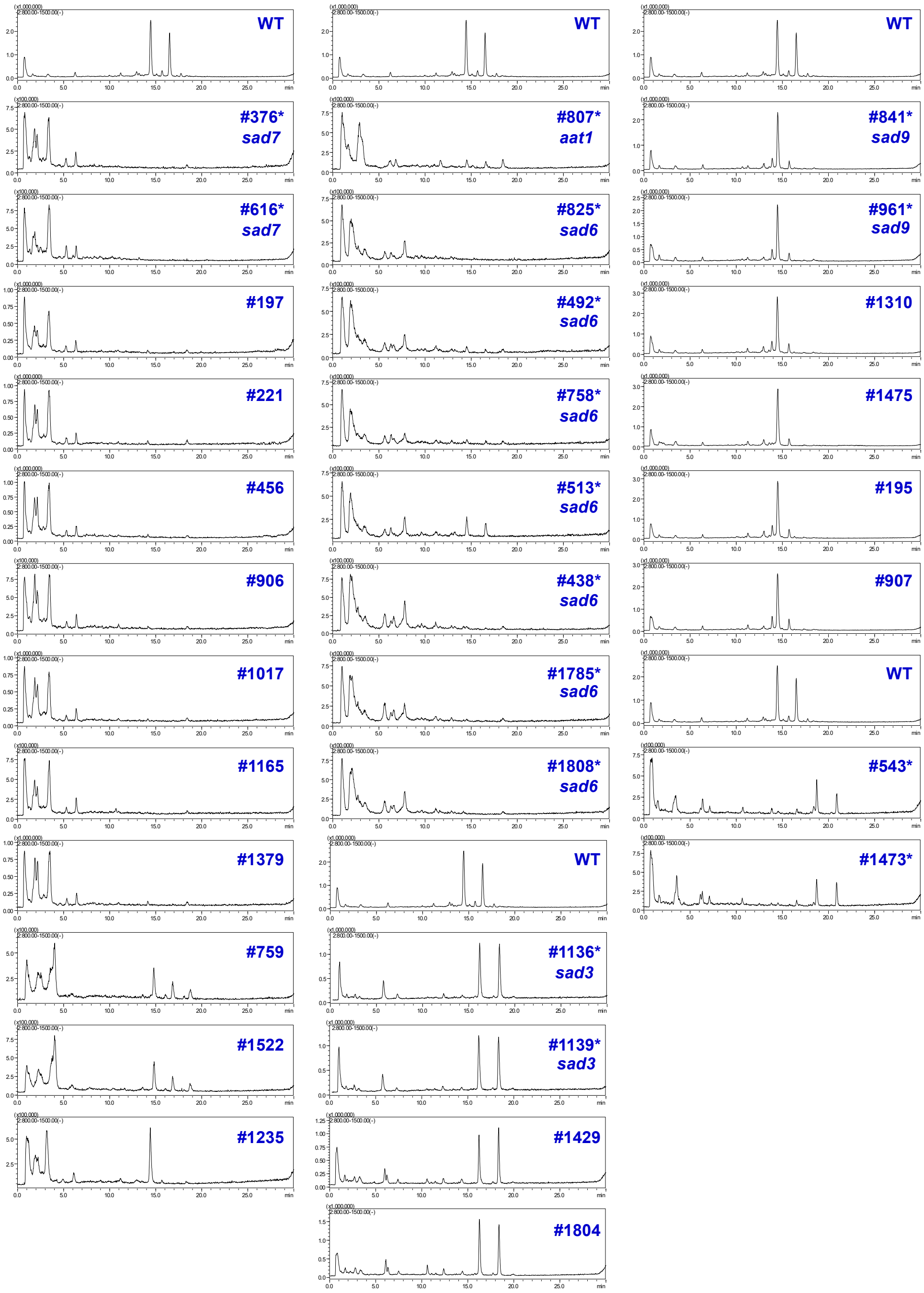

Fig. S3. Root extract chemical profiles (LC/MS) for SAD mutants that grouped together with mutants representing previously characterized loci.

*, previously characterized loci (19,20,22-24). Analytical methods described in 'Targeted LC/MS analysis and data analysis'. The mutants include for *SCPL1*/*Sad7* (10 new mutants), *MT1*/*Sad9* (4 new mutants) and *TG1/Sad3* (2 new mutants), *CYP72A475/Sad6* (0 new mutants), *AAT1* (0 new mutants), *UGT91G6* (0 new mutants).

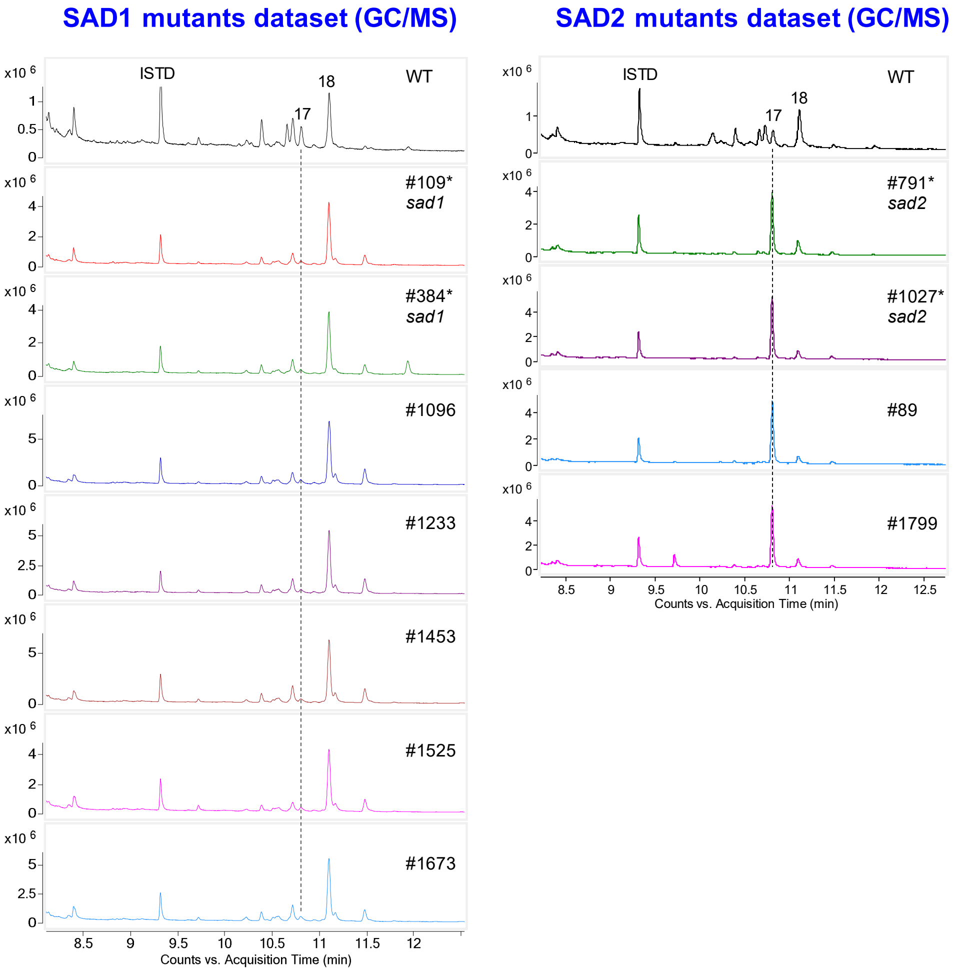

Fig. S4. Root extract chemical profiles (GC/MS) for SAD mutants that grouped together with mutants representing previously characterized loci.

*, previously characterized loci (16,17). Analytical methods described in 'GC/MS analysis'. The mutants include *bAS1/Sad1* (5 new mutants) and *CYP51H10/Sad2* (2 new mutants).

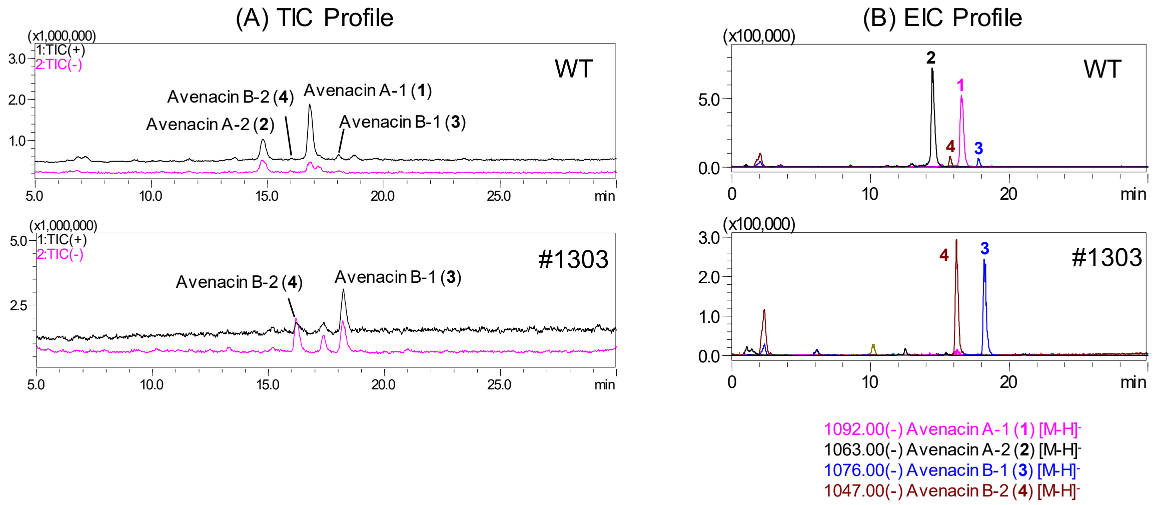

Fig. S5. Root extract chemical profile of mutant #1303 (*cyp94d65*) compared to the wild-type oat.

(A) Total ion current chromatograms. (B) Extracted ion current chromatograms for the four avenacins. Analytical methods described in ‘Targeted LC/MS analysis and data analysis’.

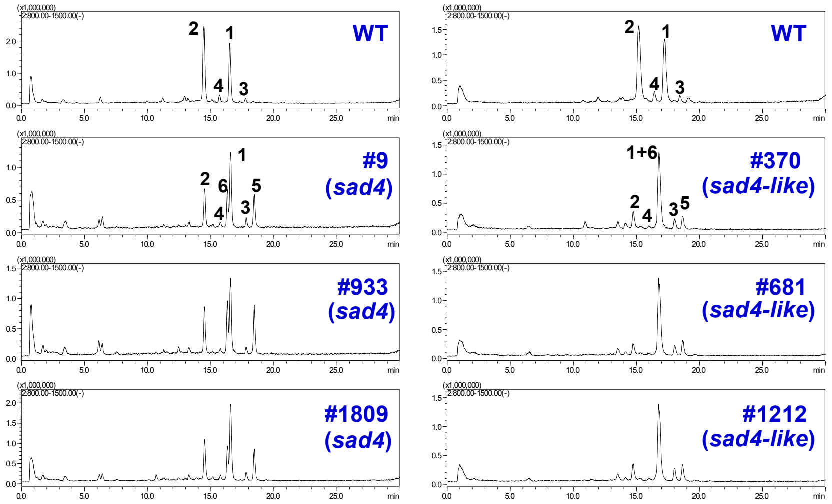

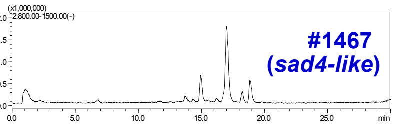

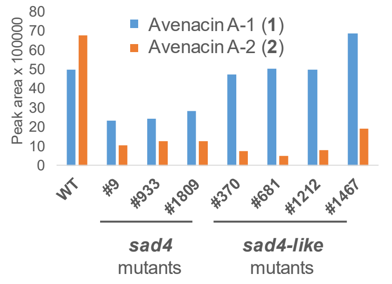

Compound No.	Name
1	Avenacin A-1
2	Avenacin A-2
3	Avenacin B-1
4	Avenacin B-2
5	Monodeglucosyl avenacin A-1
6	Monodeglucosyl avenacin A-2

Fig. S6. Root extract LC/MS chromatograms of *sad4* and *sad4-like* mutants and peak areas for avenacins A-1 (**1**) and A-2 (**2**).

Analytical methods described in ‘Targeted LC/MS analysis and data analysis’.

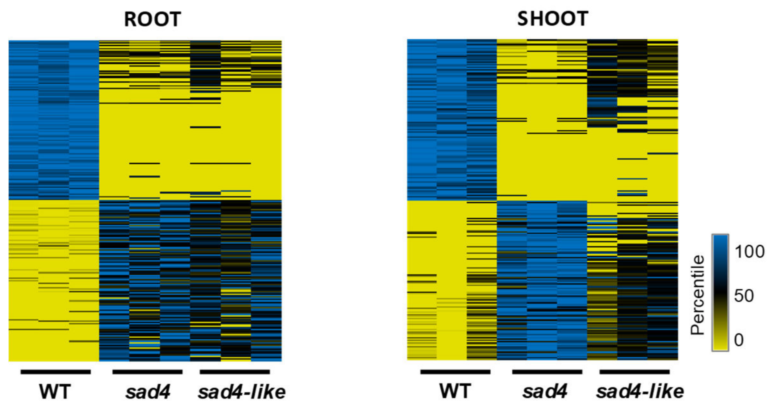

Fig. S7. Heat maps of the relative peak areas of the top 240 significantly changed compounds in roots and leaves of wild-type, *sad4* and *sad4-like* mutants (12-d-old plants).

Analytical methods described in ‘Untargeted LC/MS analysis and data analysis’.

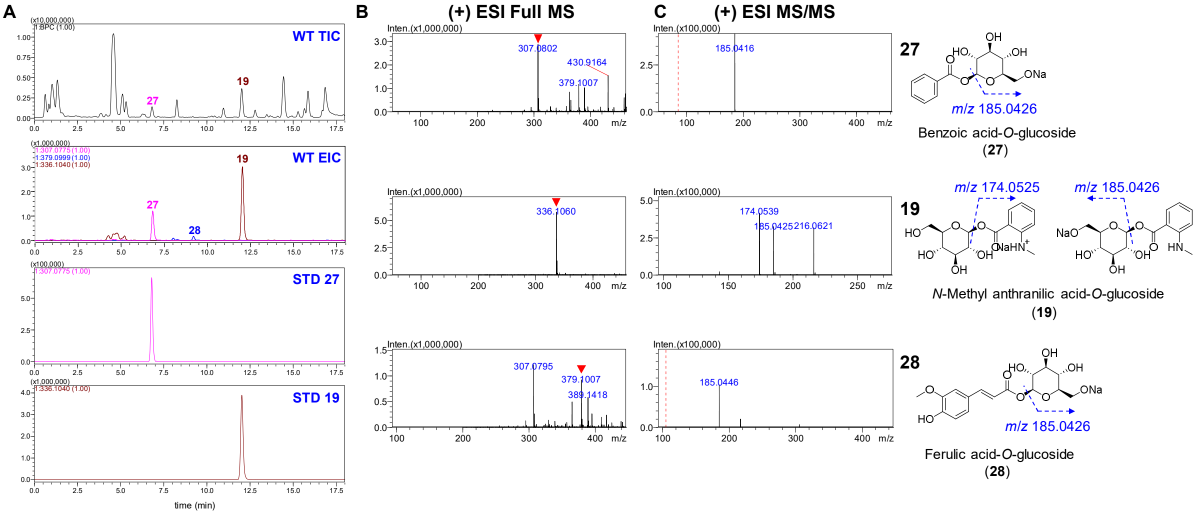

Fig. S8. Identification of acyl sugars **19**, **27**, and **28** in the root extract of wild-type oat.

(A) Chromatograms of root extract and reference standards. WT, wild-type; STD, reference standard. (B) HR-MS spectra for acyl sugars in the root extract. (C) HR-MS/MS spectra for acyl sugars in the root extract.

Analytical methods described in ‘Untargeted LC/MS analysis and data analysis’. The identity of compounds **19** and **27** were characterized by comparing with reference standards. Compound **28** was tentatively identified by analysing its MS and MS/MS spectra.

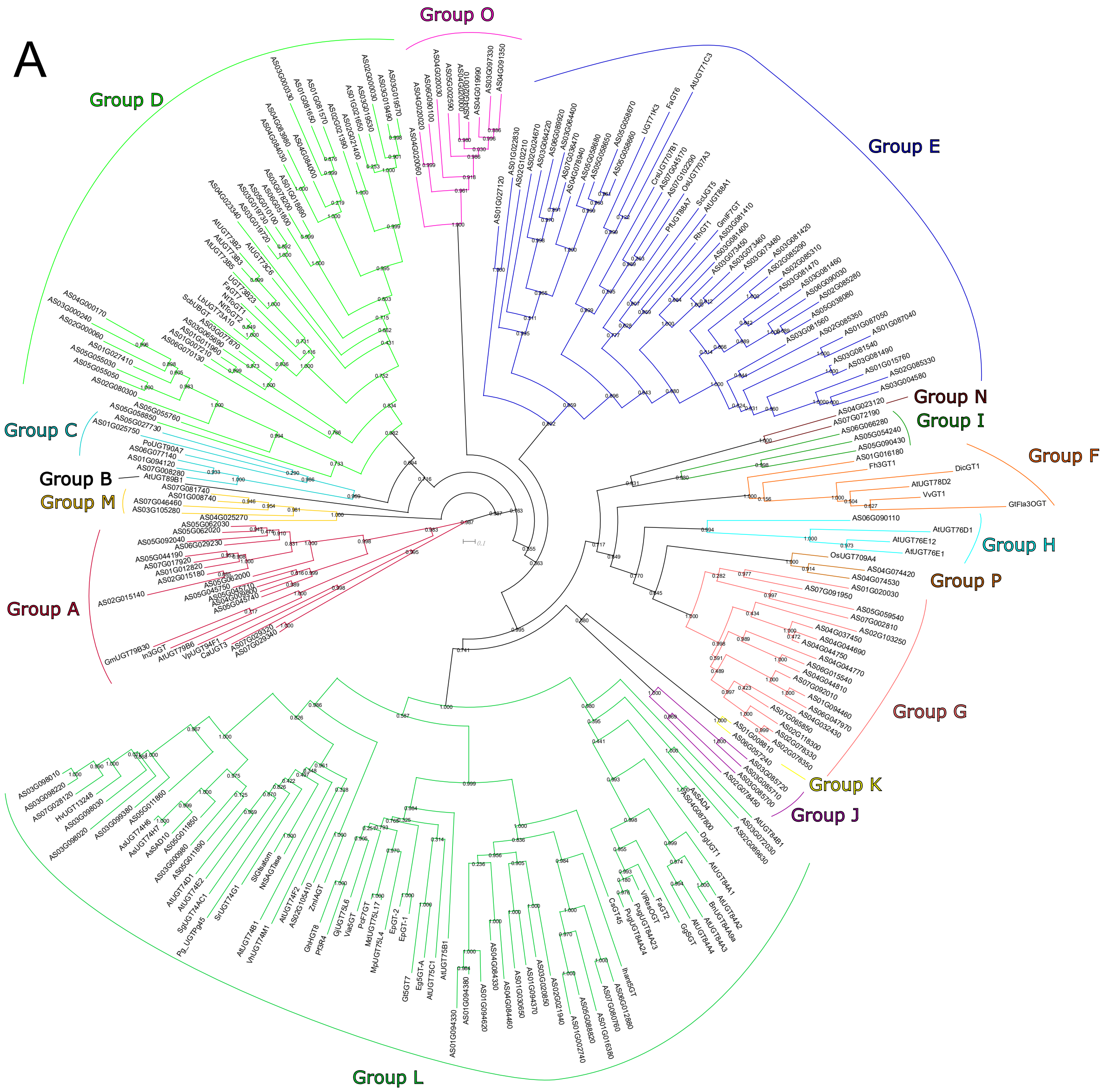

Fig. S9. Phylogenetic analysis of glycosyltransferases in oat transcriptome (A) (above) and expression pattern of group L genes (B) (below).

Accession numbers see *SI Appendix* Table S3. Alignments were done with Muscle with the option -maxiters 100. FastTree using default parameters were used. The heatmap data throughout is DeSeq normalised estimates of read counts. *, the four candidate genes selected in this study; #, previously characterised cluster genes.

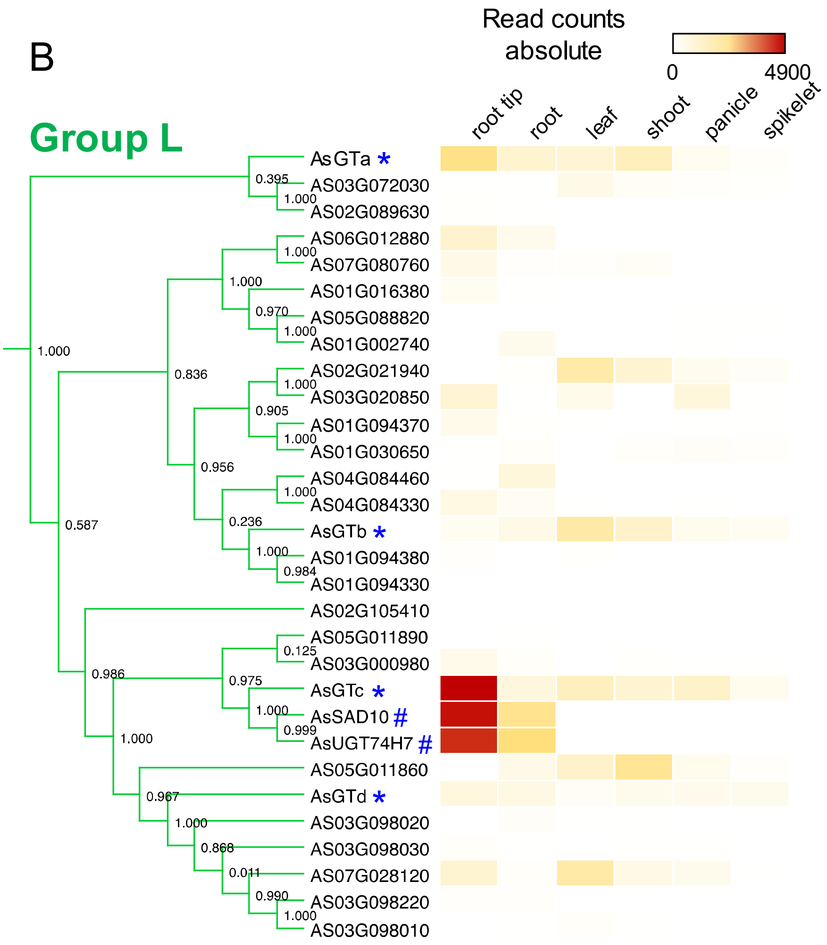

Fig. S9 (continued). Phylogenetic analysis of glycosyltransferases in oat transcriptome (A) and expression pattern of group L genes (B).

Accession numbers see *SI Appendix* Table S3. Alignments were done with Muscle with the option -maxiters 100. FastTree using default parameters were used. *, the four candidate genes selected in this study; #, previously characterised cluster genes.

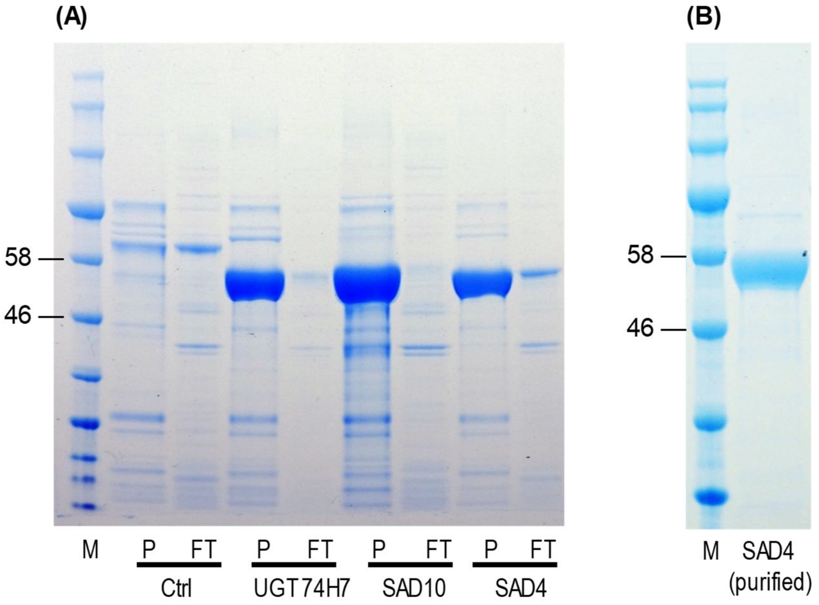

Fig. S10. SDS-PAGE of recombinant His-tagged UGT proteins.

(A) Recombinant enzymes purified using Ni-NTA agarose, used for substrate screening and comparison. (B) Recombinant SAD4 (predicted MW, 51.2 kDa) purified by affinity chromatography, used for kinetic analysis. M: Protein Marker; P, purified protein; FT, flow-through.

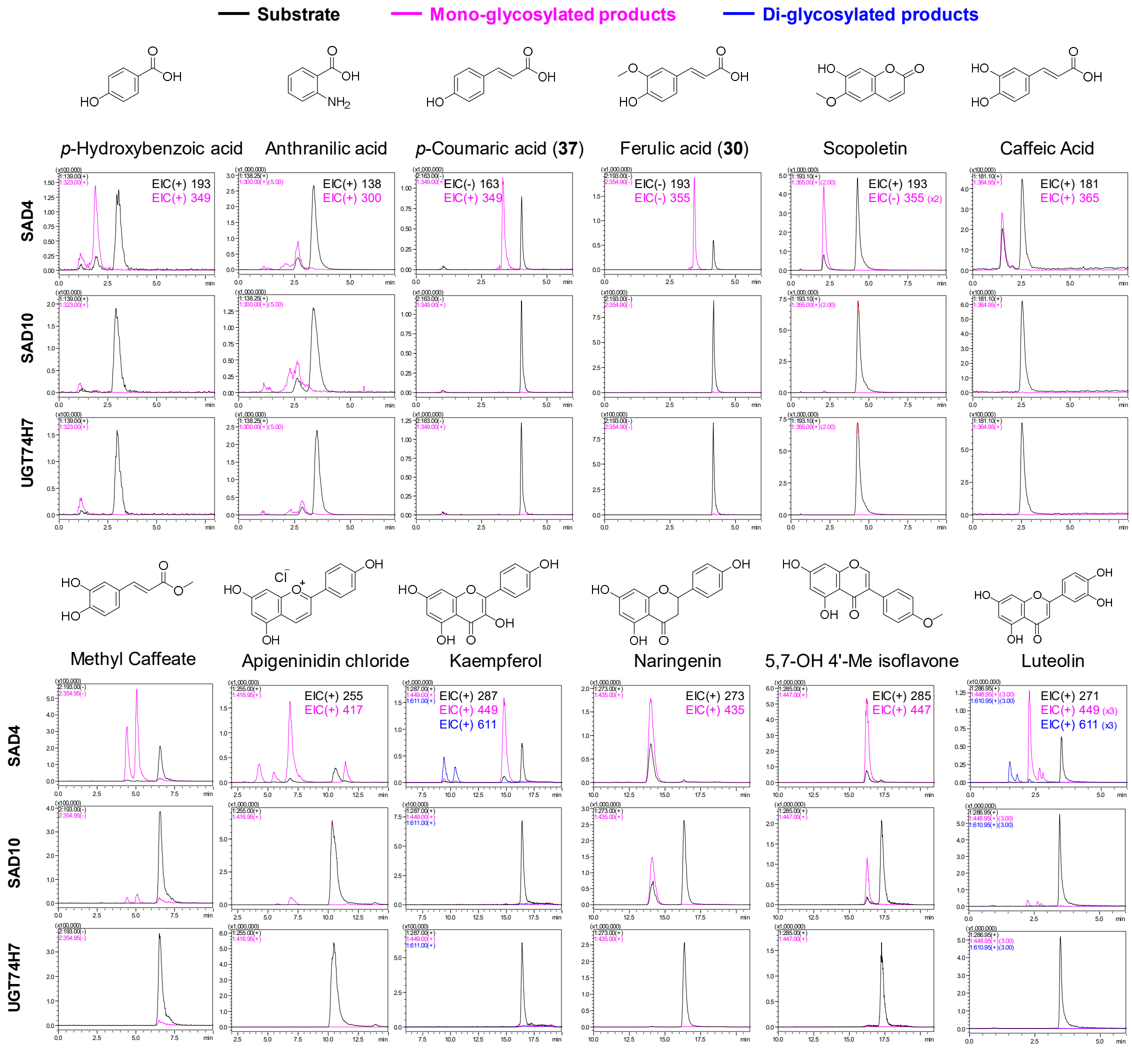

Fig. S11-1. Chromatograms of the glycosylation reaction mixtures for SAD4, SAD10, and UGT74H7 (LC/MS).

Analytical methods described in 'Glucosyltransferase activity assays'. Extracted ion chromatograms were shown for products and substrates. The extracted mass-to-charge ratio (*m*/*z*) was labelled in each panel. EIC, extracted ion chromatogram; (+) ESI positive ion mode; (-) ESI negative ion mode.

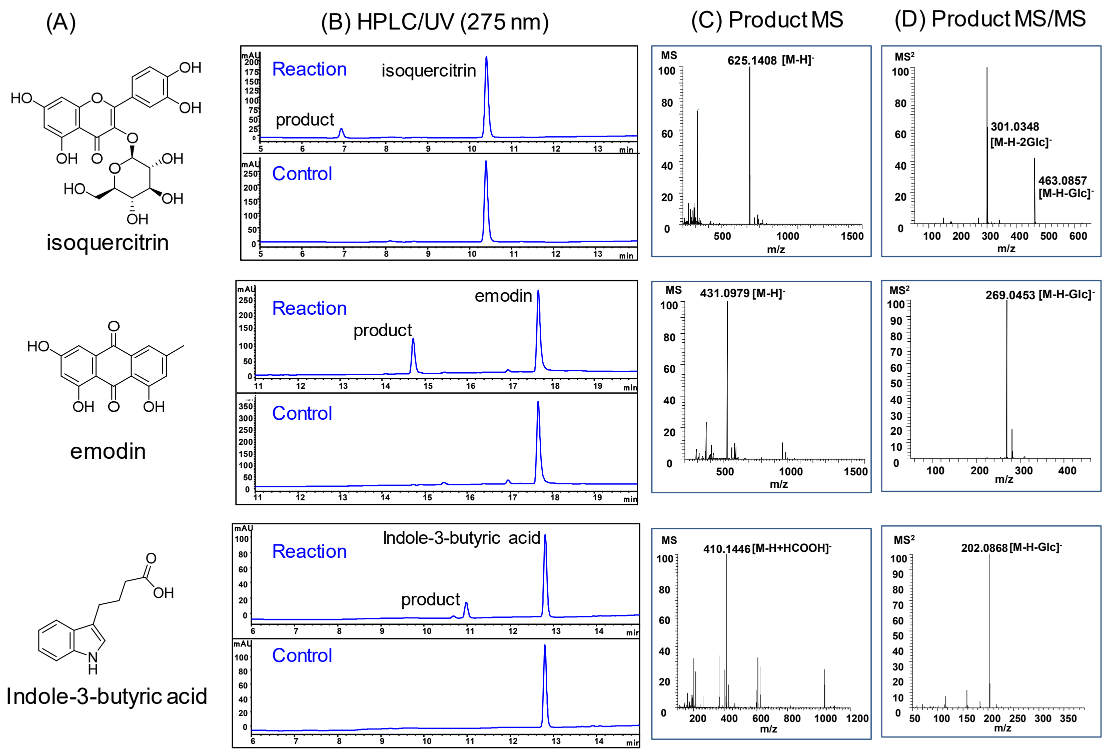

Fig. S11-2. Chromatograms of the glycosylation reaction mixtures for SAD4 (LC/UV).

(A) Structures of the substrates. (B) HPLC/UV chromatograms at 275 nm. (CD) MS and MS/MS spectra of the glycosylated products. Analytical methods described in *SI Appendix* Supplement 4. Control was conducted in the same condition reactions with the addition of boiled SAD4 protein.

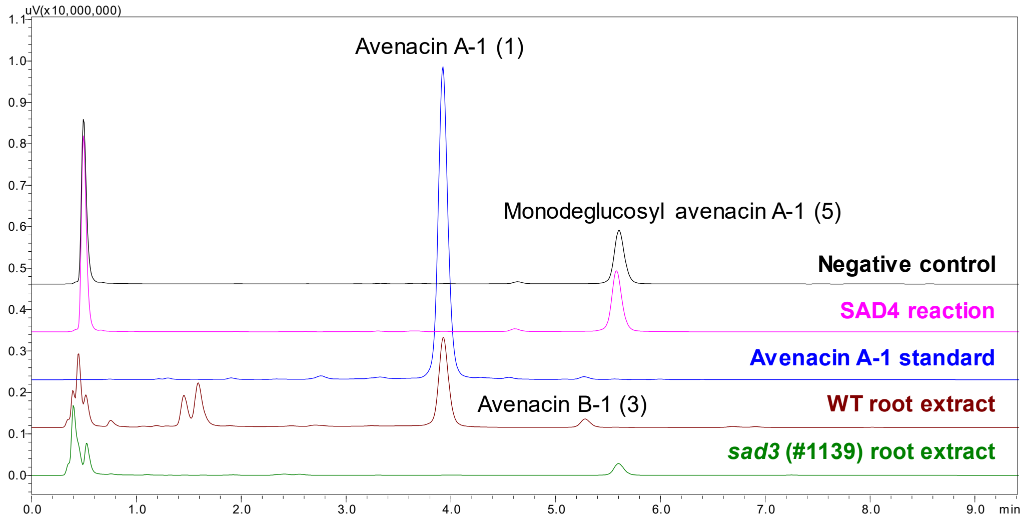

Fig. S12. SAD4 does not catalyse the glycosylation of monodeglucosyl avenacin A-1.

An enzymatic assay was conducted using SAD4 and monodeglucosyl avenacin A-1 as the substrate (see 'Glucosyltransferase activity assays' for details). Negative control was conducted in the same condition reactions with the addition of boiled SAD4. Root extracts of wild-type oat (WT) and *sad3* mutant (#1139) were used as references. Analytical methods described in the second paragraph of 'Determination of kinetic parameters for SAD4' in *SI Appendix* Supplement 2.

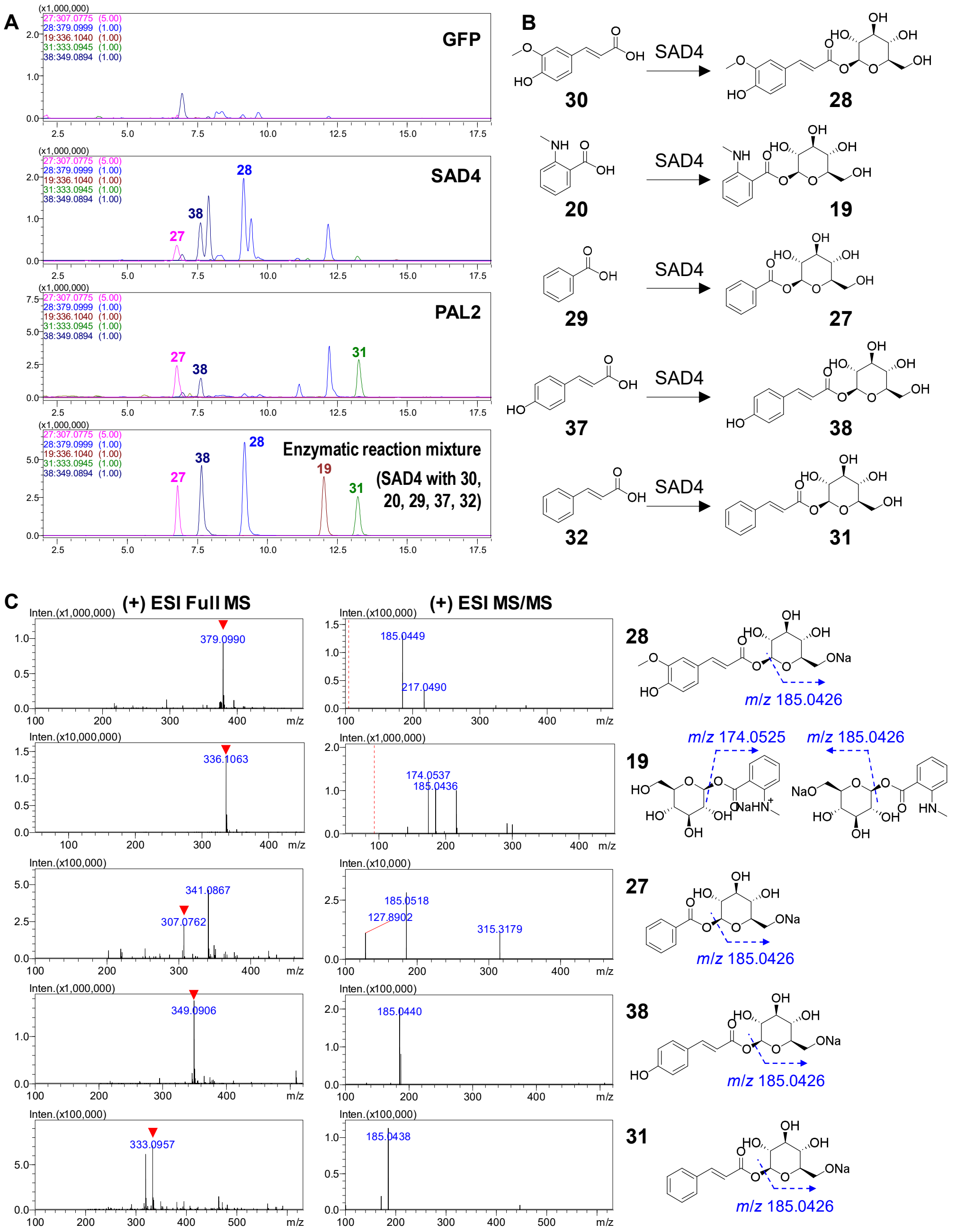

Fig. S13. Heterologous expression of SAD4 and PAL2 in *Nicotiana benthamiana*.

(A) Extracted ion chromatograms of *N. benthamiana* extracts from agroinfiltrated leaves and enzymatic reaction mixture form SAD4. GFP, green fluorescent protein. The extracted *m*/*z* values were shown in each chromatogram. (B) HR-MS and HR-MS/MS spectrums for the acyl sugars produced from enzymatic reaction (using SAD4 and compounds **30**, **20**, **29**, **37**, **32** as substrates).

Analytical methods described in 'Heterogeneous expression in *Nicotiana benthamiana*'.

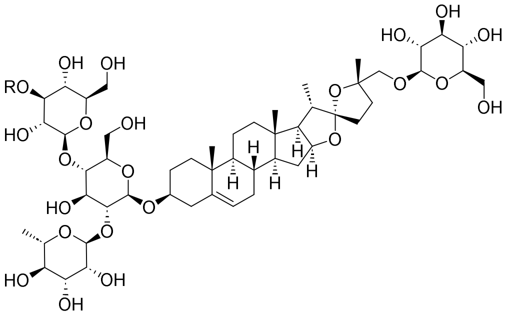

Compound name	R
Avenacoside A	H
Avenacoside B	Glc

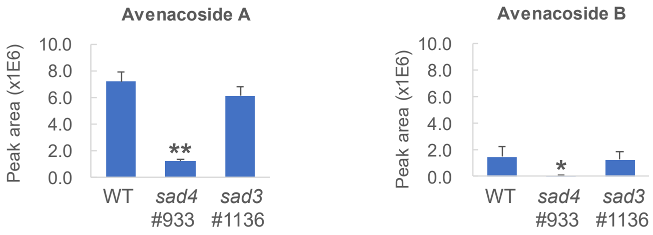

Fig. S14. Biosynthesis of the foliar steroidal saponins avenacosides A and B is also affected in *sad4* mutants.

Metabolites were extracted from 2-month-old oat leaves. Mutants of *sad3* (expressed in root tip only) were used as a negative control. Aliquots of freeze-dried leaves (5 mg) were extracted in 800 μL of methanol for 1 h. Other extraction steps were the same as for 'Targeted LC/MS analysis and data processing'. Analytical methods described in 'Targeted LC/MS analysis and data analysis'. * p<0.05, ** p<0.01 comparing to the wild type (Student's t-test).

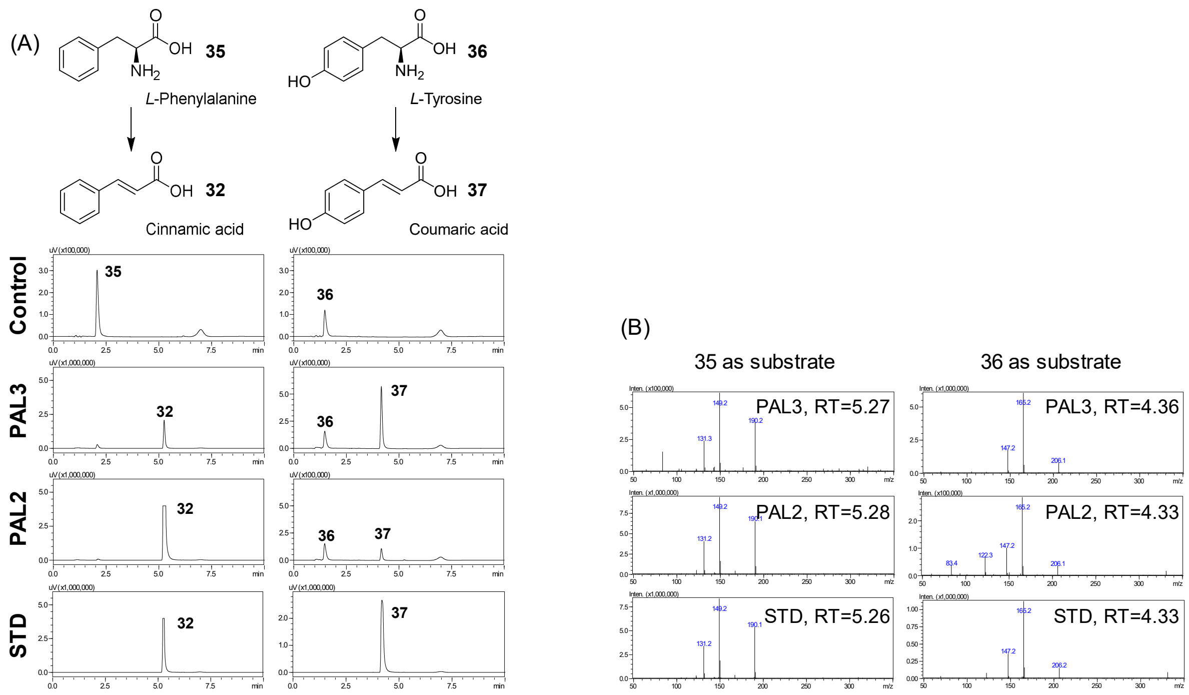

Fig. S15. Functional characterization of PAL2 and PAL3 *in vitro*.

(A) Reaction scheme and chromatogram of *in vitro* functional analysis of PAL2/3. Control, reactions with the addition of boiled PAL2/3. STD, reference standards. (B) MS/MS spectra for reaction products and reference standards.

Analytical methods described in ‘Phenylalanine ammonia-lyases activity assays’.

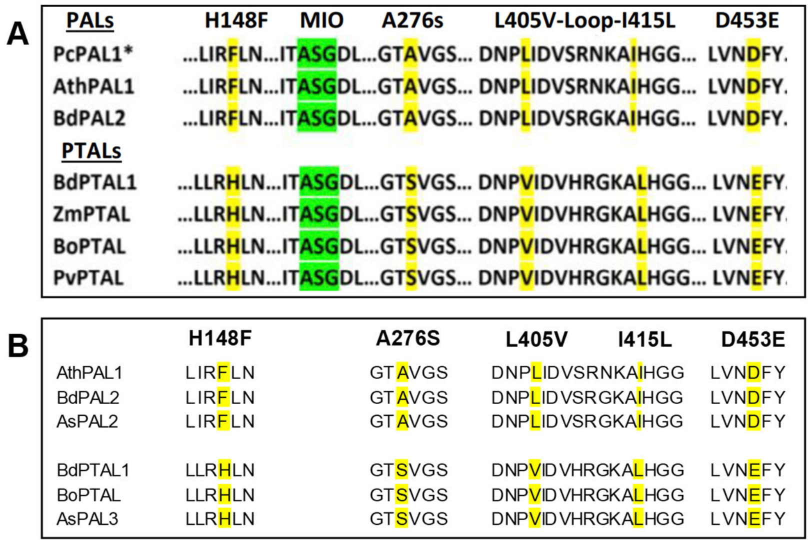

Fig. S16. Sequence alignment of PAL2 and PAL3 with other PAL and PTAL enzymes.

(A) Sequence alignment of PAL and PTAL enzymes, adapted from Figure 2B of ref. #40. (B) Sequence alignment of PAL2 and PAL3 with other characterised PAL and PTAL enzymes.

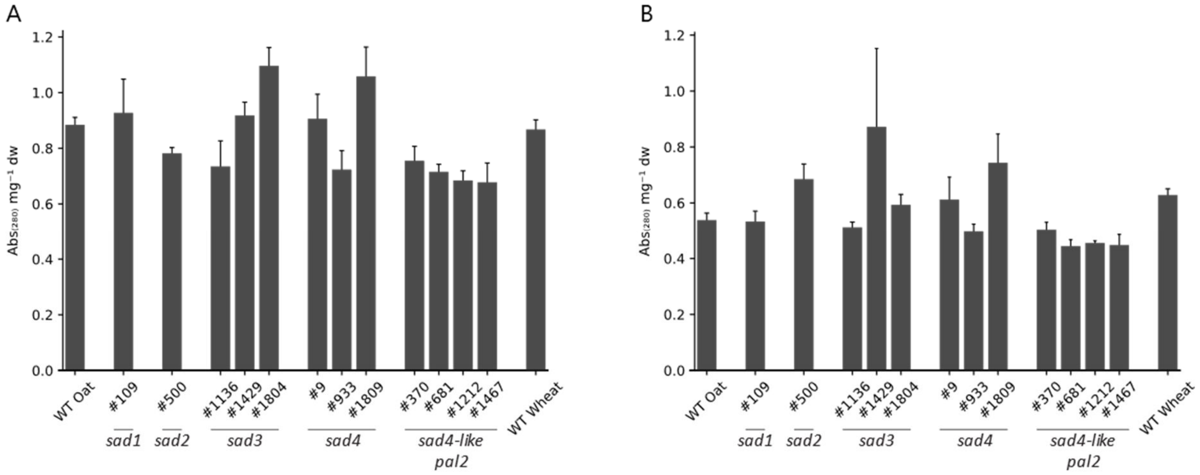

Fig. S17. Lignin quantification in wild type and mutant oat lines.

Absorbance of lignin at 280 nm for root (A) and leaf (B) tissue. WT oat, *Avena strigosa* accession S75; WT Wheat, *Triticum aestivum* L cv. Fielder. Absorbance measurements were normalised to tissue sample mass. Mean absorbance values (±SE) for n=5 replicates are shown. One-way ANOVA and post hoc Bonferroni comparisons between each mutant line and WT oat show no significant difference for either tissue type.

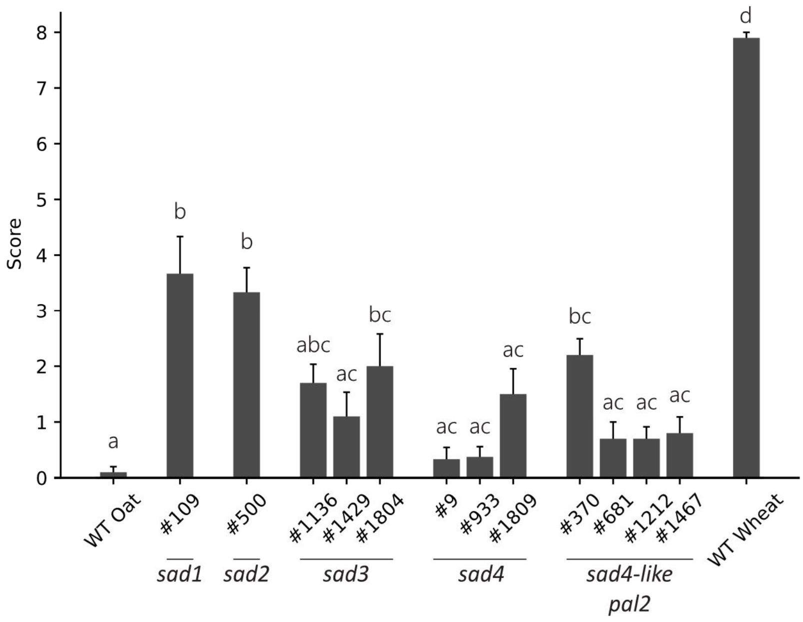

Fig. S18. Disease scores for wild type and mutant oat lines.

Mean pathogenicity scores (± SD, n= 6-10). WT Oat, *Avena strigosa* accession S75; WT Wheat, *Triticum aestivum* L cv. Fielder. Details of previously characterized mutants are shown in *AI Appendix* Table S1. Different superscript letters indicate significant differences between the groups (one-way ANOVA, post hoc ANOVA testing for all column pairs using the Bonferroni method, $P < 0.05$).

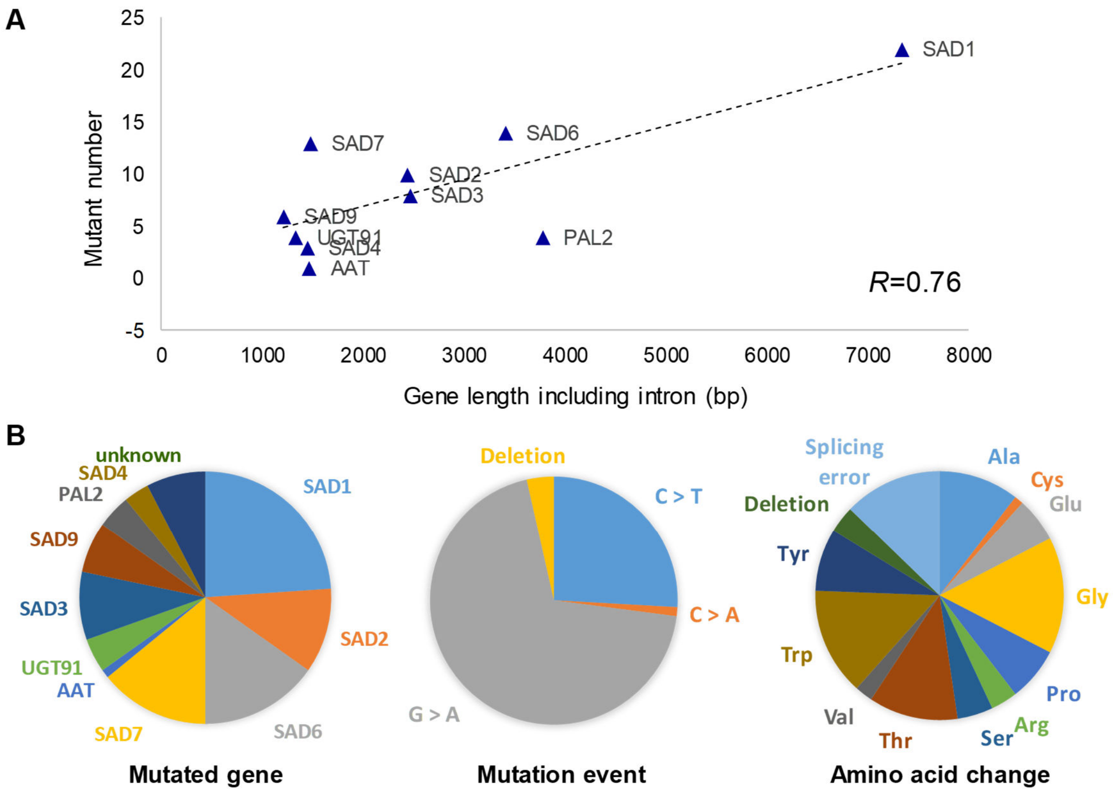

Fig. S19. Rescoring of the saponin-deficient oat mutant collection.

(A) Gene length *versus* the number of mutants characterized for that complementation group, showing a linear correlation of *R*=0.76 (R^2=0.5701); (B) Pie charts for mutation event and mutated genes.

To date, we have analyzed a total of 93 independent mutants in this library, with 86 of them showing single nucleotide variations (SNVs) in biosynthetic genes. Among the identified mutants, the majority of mutations were G>A transitions (70%) and C>T transitions (26%). The most frequent amino acid changes observed were Gly (15%), Trp (15%), splicing errors (13%), Thr (12%), and Ala (10%). Furthermore, we observed a linear correlation (*R*=0.76) between gene length and the number of mutants, which could be a valuable tool for estimating the number of mutants in TILLING populations.

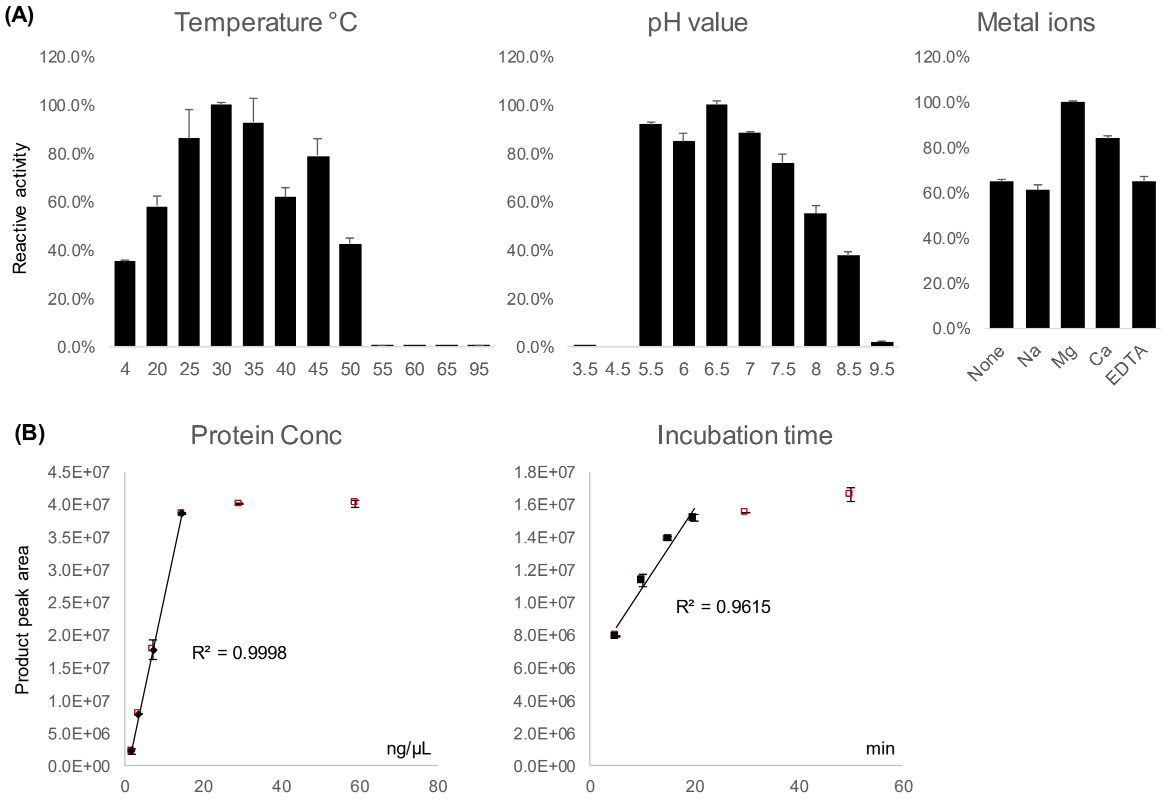

Fig. S20. Optimization of glycosylation reactions for SAD4.

(A) Effects of different temperature, pH, and metal ions to the activity of SAD4. (B) Effects of different protein concentration and incubation time to the production of glucosides. *N*-methyl anthranilic acid was used as the substrate in all reactions.

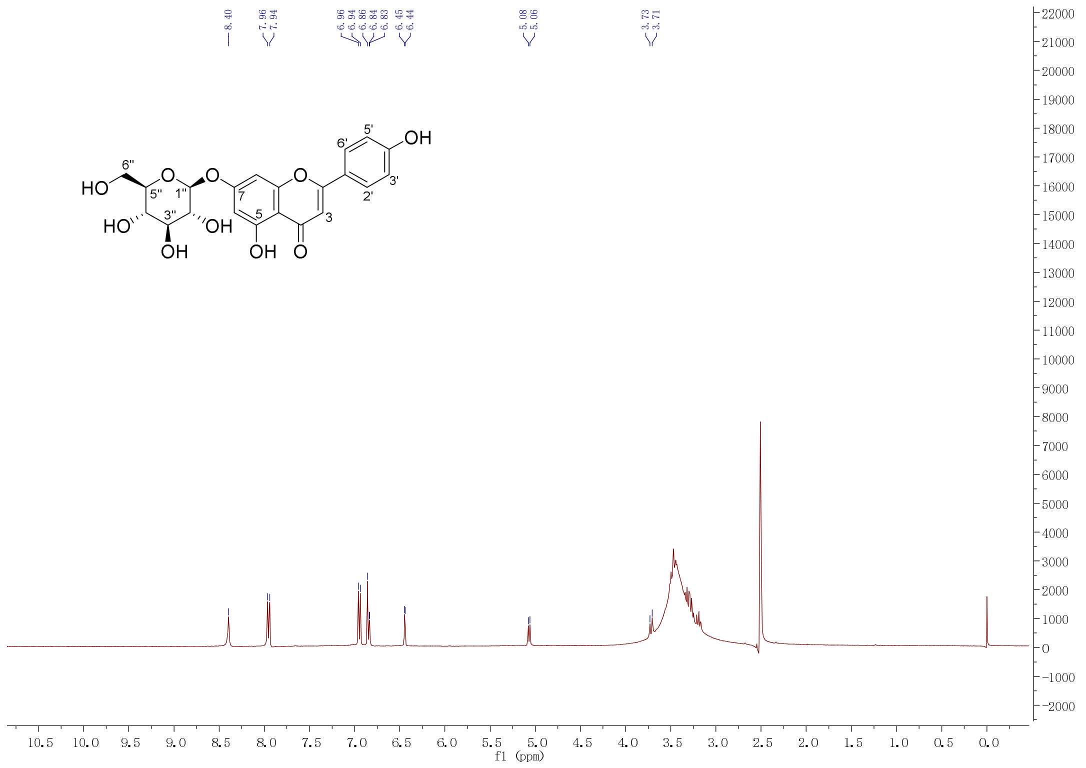

Fig. S21. ^{1}H NMR spectrum of apigenin 7-*O*-*β*-D-glucoside (**33**) in DMSO-*d*6 (400 MHz).

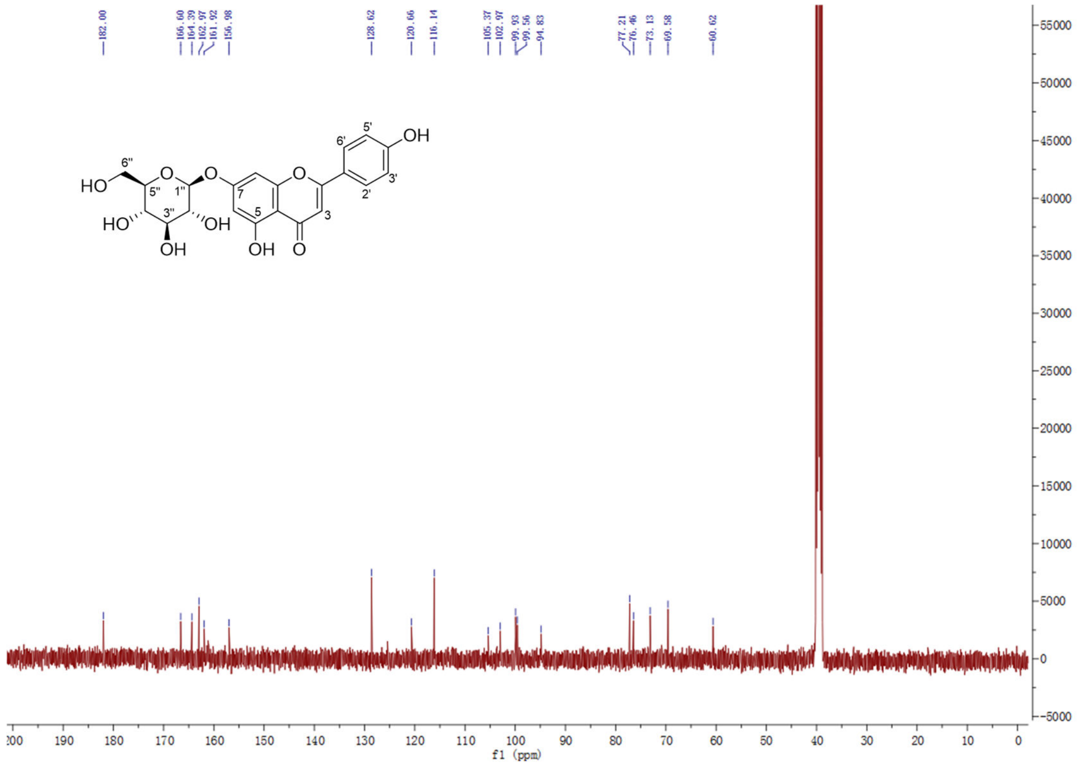

Fig. S22. ^{13}C NMR spectrum of apigenin 7-*O*-*β*-D-glucoside (**33**) in DMSO-*d*6 (400 MHz).

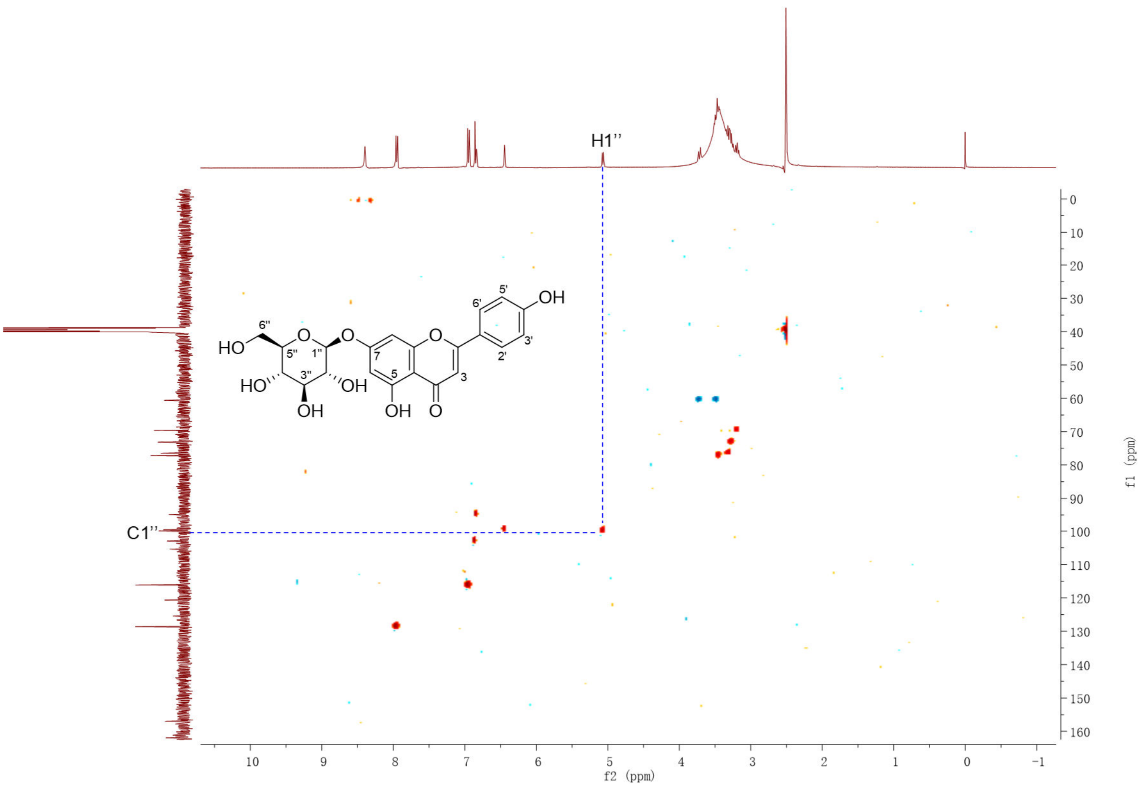

Fig. S23. HSQC spectrum of apigenin 7-*O*-*β*-D-glucoside (**33**) in DMSO-d_6 (400 MHz).

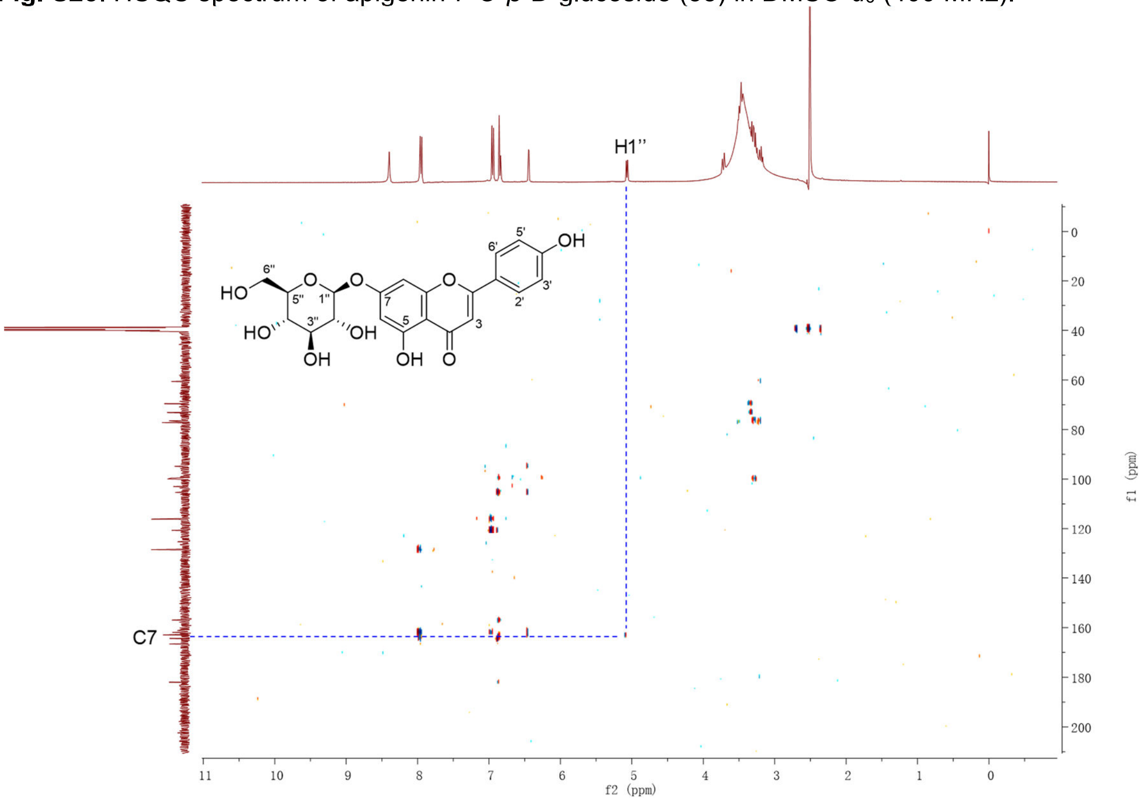

Fig. S24. HMBC spectrum of apigenin 7-*O*-*β*-D-glucoside (**33**) in DMSO-d_6 (400 MHz).

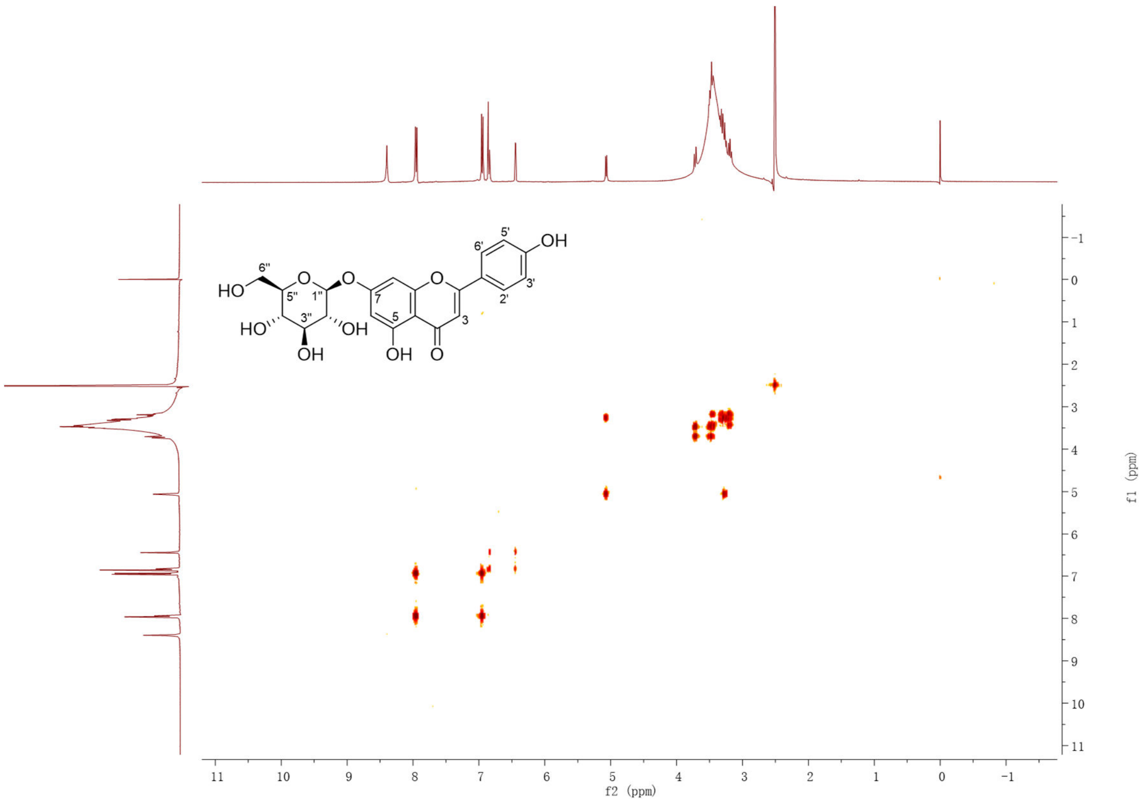

Fig. S25. H^1-H^1 COSY spectrum of apigenin 7-*O*-*β*-D-glucoside (**33**) in DMSO-d_6 (400 MHz).

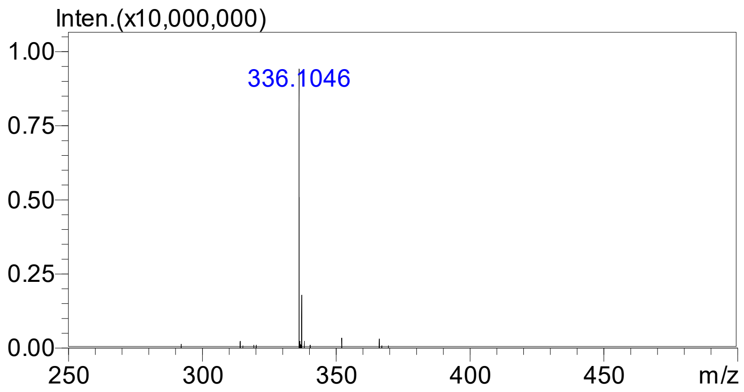

Fig. S26. HR-MS spectrum of *N*-methyl anthranilic acid *O*-glucoside (**19**).

Table S1. A full list of the saponin deficient (*sad*) oat mutant collection.

Gene	Length (ATG-stop)	Introns	Mutants	SNP	Effect	Ref
Sad1	7340 bp	17	Total 22			(16,27)
oxidosqualene cyclase			A1	G1912A	Tyr165 Stop	
			B1	G1912A	Tyr165 Stop	
			109*	G3417A	Tyr380 Stop	
			610	G1912A	Tyr165 Stop	
			1146	G4169A	Tyr471 Stop	
			1293	G39A	Tyr 13 Stop	
			110	G6689A	Splice site	
			225	G3302A	Splice site	
			589	G3914A	Splice site	
			1001	G4365A	Splice site	
			297	G3939A	Glu 419 Lys	
			358	G5234A	Cys 563 Tyr	
			384*	C7249T	Ser 728 Phe	
			532	G549A	Gly 121 Glu	
			599	G2809A	Gly 277 Glu	
			1023	C7249T	Ser 728 Phe	
			1217	G2025A	Gly 203 Glu	
			1096#	G3196A	Trp337 Stop	
			1673#	G4196A	Trp480 Stop	
			1453#	G2879A	Trp300 Stop	
			1233#	G5630A	Glu667 Lys	
			1525#	G2907A	Glu310 Lys	
Sad2	2446 bp	1	Total 10			(17)
CYP51H10			283	G2277A	Gly435 Asp	
			500	G475A	Splice site	
			638	G1922A	Glu317Lys	
			698	G1670A	Ala233Thr	
			791*	C2360T	Pro463 Ser	
			1027*	C371T	Ala124 Val	
			1325	C1866T	Ser298Phe	
			1412	C338T	Thr113Ile	
			89#	G2009A	Glu346 Lys	
			1799#	A2241 del	frame (423>)	
Sad6	3410 bp	3	Total 14			(23)
CYP72A475			825*	G2772A	T356 stop	
			1243	Deletion		
			121	G3279A	G493R	
			215	G736A	W216 stop	
			405	G3279A	G493R	
			686	G3143A	W447 stop	
			708	G2772A	W356 stop	
			1088	G2501A	Splice site	
			492*	G788A	G150D	
			758*	G788A	G150D	
			513*	G3576A	G479R	
			438*	C3424T	P428L	
			1785*	858_872del	256_260del	
			1808*	G1013A	W195 stop	
Sad7	1482 bp	0	Total 13			(19)
serine carboxypeptidase-like acyltransferase			19	C1388T	T463I	
			376*	C236T	P79L	
			616*	C410T	S137F	
			197#	C1427T	P476L	
			221#	G622A	G208R	
			456#	G536A	G179D	
			906#	G1403A	G468D	
			1017#	C1427T	P476L	
			1165#	C1218A	Y406 stop	

			1379#	G239A	G80D	
			759#	C277T	P93S	
			1522#	G256A	A86T	
			1235#	C881T	P294L	
Sad9	1210 bp	1	Total 6			(20)
N-methyltransferase			841*	C998T	A333V	
			961*	C89T	S30F	
			1310#	G978A	W326 stop	
			1475#	C235T	R79W	
			195#	G1119A	R321Q	
			907#	G787A	Splice site	
UGT91G6	1332 bp	0	Total 4			(24)
UDP-glucosyltransferase			85	G963A	W321 stop	
UGT91G6			543*	G375A	W125 stop	
			1073	G776A	G259E	
			1473*	G775A	G259R	
Sad3	2465 bp	8	Total 8			(24)
transglucosidase			105	G1705A	Splice site	
			368	G216A	Val29Met	
			891	C481T	Ala88Val	
			986	G1705A	Splice site	
			1136*	G2049A	Splice site	
			1139*	G1800A	Splice site	
			1429#	G945A	Val166 Met	
			1804#	G1729A	Trp360 stop	
AAT1	1470 bp	0	Total 1			(22)
UDP-						
arabinosyltransferase			807*	G753A	W251 stop	
UGT99D1						
CYP94D65	2667 bp	1	Total 1			(15)
			1303#	G1385A	W423 stop	
Sad4	1452 bp	0	Total 3			(18)
UDP-glucosyltransferase			9#	C404T	P135L	
UGT84C2			933#	G963A	W321 stop	
			1809#	C404T	P135L	
PAL2	3782bp	1	Total 4			
L-phenylalanine			370#	G323A	G108E	
ammonia-lyase			681#	C451T	R151C	
			1212#	G409A	G137R	
			1467#	C556T	P186S	
Uncharacterized			1523			
			1607			
			964			
			1610			
			1611			
			1102			
			127			

*previously characterized mutants used as reference in this study; # mutants characterized in this study.

Table S2. Glycosyltransferase genes involved in *SI Appendix* Fig. S9.

Name	Genbank Acc. No.	Plant species	Substrate	Group	UGT family	Reference PMID or DOI
AsSAD10	ACD03250.1	*Avena strigosa*	Benzoic acid, *N*-methyl anthranilic acid	L	UGT74	23258535
AsSAD4	ACD03236.1	*Avena strigosa*	Benzoic acid, *N*-methyl anthranilic acid	L	UGT84	23258535
AsUGT74H6	ACD03261.1	*Avena strigosa*	Benzoic acid	L	UGT74	23258535
AsUGT74H7	ACD03246.1	*Avena strigosa*	Benzoic acid	L	UGT74	23258535
AtUGT71C3	NP_172206.1	*Arabidopsis thaliana*	Dihydroxybenzoic acid	E	UGT71	11641410
AtUGT73B2	NP_567954.1	*Arabidopsis thaliana*	2,4,6-Trinitrotoluene	D	UGT73	18702669
AtUGT73B3	NP_567953.1	*Arabidopsis thaliana*	Flavonoid	D	UGT73	16794327
AtUGT73B5	NP_179150.3	*Arabidopsis thaliana*	2,4,6-Trinitrotoluene	D	UGT73	18702669
AtUGT73C6	NP_181217.1	*Arabidopsis thaliana*	Flavonoid	D	UGT73	12900416
AtUGT74B1	O48676.1	*Arabidopsis thaliana*	Glucosinolate	L	UGT74	15584955
AtUGT74D1	Q9SKC5.1	*Arabidopsis thaliana*	Auxin	L	UGT74	24285754
AtUGT74E2	NP_172059.1	*Arabidopsis thaliana*	Indole 3-butyric acid	L	UGT74	20798329
AtUGT74F2	AAB64024.1	*Arabidopsis thaliana*	Salicylic acid, benzoic acid	L	UGT74	11641410
AtUGT75B1	AEE27854.1	*Arabidopsis thaliana*	Indole acetic acid	L	UGT75	11641410
AtUGT75C1	Q0WW21.2	*Arabidopsis thaliana*	Anthocyanin	L	UGT75	15807784
AtUGT76D1	AEC07843.1	*Arabidopsis thaliana*	Flavonoid	H	UGT76	15352060
AtUGT76E1	AED97208.1	*Arabidopsis thaliana*	Flavonoid	H	UGT76	15352060
AtUGT76E12	Q94AB5.1	*Arabidopsis thaliana*	Flavonoid	H	UGT76	15352060
AtUGT78D2	Q9LFJ8.1	*Arabidopsis thaliana*	Flavonoid	F	UGT78	22249996
AtUGT79B6	Q9FN26.1	*Arabidopsis thaliana*	Flavonoid	A	UGT79	24916675
AtUGT84A1	Q5XF20.1	*Arabidopsis thaliana*	Hydroxycinnamate	L	UGT84	11187886
AtUGT84A2	Q9LVF0.1	*Arabidopsis thaliana*	Phenylpropanoid	L	UGT84	11187886
AtUGT84A3	O23401.1	*Arabidopsis thaliana*	Hydroxycinnamate	L	UGT84	11187886
AtUGT84A4	O23402.1	*Arabidopsis thaliana*	Hydroxycinnamate	L	UGT84	11187886
AtUGT84B1	NP_179907.1	*Arabidopsis thaliana*	Auxin	L	UGT84	11042207
AtUGT88A1	AEE75831.1	*Arabidopsis thaliana*	Flavonoid	E	UGT88	15352060
AtUGT89B1	NP_177529.2	*Arabidopsis thaliana*	Phenolic acid, flavonoid	B	UGT89	15352060

BnUGT84A9a	CAS03354.1	*Brassica napus* var. *napus*	Sinapate; hydroxycinnamates	L	UGT84	20087565
CaUGT3	BAH80312.1	*Catharanthus roseus*	Flavonoid	A	UGT94	19561332
CrsUGT707B1	CCG85331.1	*Crocus sativus*	Flavonoid	E	UGT707	22649274
CsGT45	ACM66950.1	*Crocus sativus*	Flavonoid	L	UGT75	19695093
DgUGT1	BAO66179.1	*Delphinium grandiflorum*	Phenolic acids	L	UGT84	24723398
DicGT1	BAD52003.1	*Dianthus caryophyllus*	Flavonoid	F	UGT78	https://doi.org/10.5511/plantbiotechnology.21.367
FaGT2	Q66PF4.1	*Fragaria x ananassa*	Cinnamic acids; benzoic acids	L	UGT84	16443693
FaGT6	Q2V6K0.1	*Fragaria x ananassa*	Flavonoid - flavonol	E	UGT71	18487633
FaGT7	Q2V6J9.1	*Fragaria x ananassa*	Flavonoid - flavonol	D	UGT73	18487633
Fh3GT1	ADK75021.1	*Freesia* hybrid cultivar	Flavonoid	F	UGT78	21318353; 27064818
GgSGT	BAG14302.1	*Gomphrena globosa*	Sinapate	L	UGT84	https://doi.org/10.5511/plantbiotechnology.25.369
GhHGT8	Q9ZR25.1	*Glandularia x hybrida*	Flavonoid	L	UGT75	10066805
GjUGT75L6	F8WKW0.1	*Gardenia jasminoides*	Apocarotenoid	L	UGT75	21799001
GmIF7GT	NP_001235161.1	*Glycine max*	Flavonoid	E	UGT88	17565994
GmUGT79B30	BAR88077.1	*Glycine max*	Flavonoid	A	UGT79	26002063
Gt5GT7	B2NID7.1	*Gentiana triflora*	Flavonoid	L	UGT75	18375606
GtFla3OGT	Q96493.1	*Gentiana triflora*	Flavonoid	F	UGT78	8819318
HvUGT13248	ADC92550.1	*Hordeum vulgare* subsp. *vulgare*	Epoxy-sesquiterpenoid	L	UGT84	20521959
Ihant5GT	BAD06874.1	*Iris x hollandica*	Anthocyanin	L	UGT75	https://doi.org/10.1016/j.plantsci.2004.06.020
In3GGT	Q53UH4.1	*Ipomoea nil*	Anthocyanidin	A	UGT91	15842621
LbUGT73A10	BAG80536.1	*Lycium barbarum*	Flavonoid	D	UGT73	https://doi.org/10.1016/j.molcatb.2008.02.001
MdUGT75L17	AAX16493.1	*Malus domestica*	Dihydrochalcone	L	UGT75	27316677
MpUGT75L4	ABL85474.1	*Maclura pomifera*	Flavonoid	L	UGT75	17157841
NtSAGTase	AAF61647.1	*Nicotiana tabacum*	Salicylic acid	L	UGT74	10593966
NtToGT1	Q9AT54.1	*Nicotiana tabacum*	Phenolic acids; coumarin	L	UGT73	9824316
NtToGT2	AAB36652.1	*Nicotiana tabacum*	Salicylic acid, scopoletin	L	UGT75	9824316

OsUGT707A3	BAC83989.1	*Oryza sativa* Japonica Group	Flavonoid	E	UGT71	17363107
OsUGT709A4	BAC80066.1	*Oryza sativa* Japonica Group	Isoflavonoid	H	UGT76	17363107
PcF7GT	AAY27090.1	*Pyrus communis*	Flavonol	L	UGT75	9167271
Pf3R4	BAA36421.1	*Perilla frutescens* var. *crispa*	Anthocyanin	L	UGT75	10066805
PfUGT88A7	BAG31949.1	*Perilla frutescens*	Flavonoid	E	UGT88	19454730
Pg_UGTPg45	AKA44586.1	*Panax ginseng*	Triterpene	L	UGT74	25769286
PoUGT90A7	ACB56926.1	*Pilosella officinarum*	Flavonoid	C	UGT90	19238428
PugUGT84A23	ANN02875.1	*Punica granatum*	Phenolic acids	L	UGT84	27227328
PugUGT84A24	ANN02877.1	*Punica granatum*	Phenolic acids	L	UGT84	27227328
RhGT1	BAD99560.1	*Rosa* hybrid cultivar	Anthocyanidin	E	UGT88	15944692
ScbUBGT	BAA83484.1	*Scutellaria baicalensis*	Flavonoid	D	UGT73	10872235
ScUGT5	BAJ11653.1	*Sinningia cardinalis*	Anthocyanidin	E	UGT88	20458497
SgUGT74AC1	AEM42999.1	*Siraitia grosvenorii*	Triterpene	L	UGT74	25759326
SlGtsatom	CAI62049.1	*Solanum lycopersicum*	5-Hydroxysalicylic acid	L	UGT74	20729481
SrUGT74G1	Q6VAA6.1	*Stevia rebaudiana*	Diterpenoid	L	UGT74	15610349
UGT71K3	XP_004294260.1	*Fragaria vesca* subsp. *vesca*	Furanone	E	UGT71	26993618
UGT73B23	XP_004304022.1	*Fragaria vesca* subsp. *vesca*	3-Hydroxycoumarin; flavonoid	L	UGT73	26859691
VhUGT74M1	ABK76266.1	*Gypsophila vaccaria*	Triterpene	L	UGT74	17172290
Via5GT	AHL68667.1	*Vitis amurensis*	Anthocyanin	L	UGT75	26159788
VlResOGT	ABH03018.1	*Vitis labrusca*	Flavonoid	L	UGT84	17270014
VpUGT94F1	BAI44133.1	*Veronica persica*	Flavonoid	A	UGT94	20223486
VvGT1	AAB81683.1	*Vitis vinifera*	Flavonoid	F	UGT78	9535914
ZmIAGT	AAA59054.1	*Zea mays*	Auxin	L	UGT74	8085154

Table S3. Analysis of F2 progeny derived from a cross between wild type and sad4 mutant #933.

Chemotype		No. of progeny	Expected genotype	No. of progeny
A-1	MDG A-1			
+	-	144	*Sad4Sad4*	43
+	-		*Sad4sad4*	99
+	+	48	*sad4sad4*	50

Segregation is consistent with a 1:2:1 ratio (P > 0.9). MDG A-1, monodeglucosyl avenacin A-1.

Table S4. Glycosyltransferase genes involved in Figure 3B.

Gene Name	Genbank Acc. No.	Species	Reported substrate	Bond type	Sugar donor	Group	UGT Family	Reference PMID or DOI
AsSAD4	ACD03236.1	*Avena strigosa*	Benzoic acid, *N*-methyl anthranilic acid	ester	UDP-Glu	L	UGT84	23258535
AsSAD10	ACD03250.1	*Avena strigosa*	Benzoic acid, *N*-methyl anthranilic acid	ester	UDP-Glu	L	UGT74	23258535
AsUGT74H6	ACD03261.1	*Avena strigosa*	Benzoic acid	ester	UDP-Glu	L	UGT74	23258535
AsUGT74H7	ACD03246.1	*Avena strigosa*	Benzoic acid	ester	UDP-Glu	L	UGT74	23258535
SlGtsatom	CAI62049.1	*Solanum lycopersicum*	5-Hydroxysalicylic acid	non-ester	UDP-Xyl	L	UGT74	20729481
NtSAGTase	AAF61647.1	*Nicotiana tabacum*	Salicylic acid	non-ester	UDP-Glu	L	UGT74	10593966
AtUGT74D1	Q9SKC5.1	*Arabidopsis thaliana*	Auxin	ester	UDP-Glu	L	UGT74	24285754
AtUGT74E2	NP_172059.1	*Arabidopsis thaliana*	Indole 3-butyric acid	ester	UDP-Glu	L	UGT74	20798329
ZmIAGT	AAA59054.1	*Zea mays*	Auxin	ester	UDP-Glu	L	UGT74	8085154
AtUGT84B1	NP_179907.1	*Arabidopsis thaliana*	Auxin	ester	UDP-Glu	L	UGT84	11042207
DgUGT1	BAO66179.1	*Delphinium grandiflorum*	Phenolic acids	ester	UDP-Glu	L	UGT84	24723398
PugUGT84A23	ANN02875.1	*Punica granatum*	Phenolic acids	ester	UDP-Glu	L	UGT84	27227328
GgSGT	BAG14302.1	*Gomphrena globosa*	Sinapate	ester	UDP-Glu	L	UGT84	DOI: 10.5511/plantbiotechnology.25.369
AtUGT84A1	Q5XF20.1	*Arabidopsis thaliana*	Hydroxycinnamate	ester	UDP-Glu	L	UGT84	11187886
AtUGT84A2	Q9LVF0.1	*Arabidopsis thaliana*	Phenylpropanoid	ester	UDP-Glu	L	UGT84	11187886
AtUGT84A3	O23401.1	*Arabidopsis thaliana*	Hydroxycinnamate	ester	UDP-Glu	L	UGT84	11187886
AtUGT84A4	O23402.1	*Arabidopsis thaliana*	Hydroxycinnamate	ester	UDP-Glu	L	UGT84	11187886
BnUGT84A9a	CAS03354.1	*Brassica napus* var. *napus*	Sinapate; hydroxycinnamates	ester	UDP-Glu	L	UGT84	20087565
NtToGT1	Q9AT54.1	*Nicotiana tabacum*	Phenolic acids; coumarin	non-ester	UDP-Glu	L	UGT73	9824316
NtToGT2	AAB36652.1	*Nicotiana tabacum*	Salicylic acid, scopoletin	non-ester	UDP-Glu	L	UGT75	8843948
UGT73B23	XP_004304022.1	*Fragaria vesca* subsp. *vesca*	3-Hydroxycoumarin; flavonoid	non-ester	UDP-Glu	L	UGT73	26859691
AtUGT73B2	NP_567954.1	*Arabidopsis thaliana*	2,4,6-Trinitrotoluene	non-ester	UDP-Glu	D	UGT73	18702669

AtUGT73B3	NP_567953.1	*Arabidopsis thaliana*	Flavonoid	non-ester	UDP-Glu	D	UGT73	16794327
AtUGT71C3	NP_172206.1	*Arabidopsis thaliana*	Dihydroxybenzoic acid	non-ester	UDP-Glu	E	UGT71	11641410
AtUGT73B5	NP_179150.3	*Arabidopsis thaliana*	2,4,6-Trinitrotoluene	non-ester	UDP-Glu	D	UGT73	18702669

Table S5. Kinetic parameters for SAD4, SAD10 and UGT74H7 towards different substrates.

		K_m mM	V_m mM s^{-1}	k_{cat} s^{-1}	k_{cat}/K_m s^{-1} mM^{-1}
UGT84C2 (SAD4)[a]	Salicylic acid	-	-	-	-
	Benzoic acid	0.1794 ± 0.0287	2.64E-05	0.046	0.255
	N-Methyl anthranilic acid	0.0068 ± 0.0026	7.29E-06	0.051	7.426
	Apigenin	0.0398 ± 0.0010	6.27E-05	0.123	3.100
	Cinnamic acid [b]	0.0662 ± 0.0208	3.22E-05	0.056	0.844
UGT74H5 (SAD10)[c]	Salicylic acid	-	-	-	-
	Benzoic acid	0.269 ± 0.046	6.70E-06	0.045	0.167
	N-Methyl anthranilic acid	0.025 ± 0.005	1.16E-05	0.075	3.000
	Anthranilic acid	0.421 ± 0.101	2.17E-05	0.140	0.333
UGT74H7[c]	Salicylic acid	-	-	-	-
	Benzoic acid	0.330 ± 0.096	8.00E-06	0.051	0.154
	N-Methyl anthranilic acid	-	-	-	-
	Anthranilic acid	1.857 ± 0.380	1.80E-06	0.014	0.006

[a] Values for SAD4 are the means ± SD (*n*=3).
[b] Concentration of cinnamic acid glucoside were calculated using the calibration curve of cinnamic acid under the wavelength of 275 nm.
[c] Values for SAD10 and UGT74H7 were adopted from the previous report (21).
-, no activity detected.

Table S6. Phenylalanine ammonia-lyase genes involved in Figure 4A.

Name	Genbank Acc. No.	Plant species	Function	PMID
AtPAL1	AY303128	*Arabidopsis thaliana*	phenylalanine ammonia-lyase	15276452
AtPAL2	AY303129	*Arabidopsis thaliana*	phenylalanine ammonia-lyase	15276452
AtPAL3	AY528562	*Arabidopsis thaliana*	phenylalanine ammonia-lyase	15276452
AtPAL4	AY303130	*Arabidopsis thaliana*	phenylalanine ammonia-lyase	15276452
BdPTAL1	XM_003575348.1	*Brachypodium distachyon*	bifunctional ammonia-lyase	27255834
BdPAL2	XM_003575352.1	*Brachypodium distachyon*	phenylalanine ammonia-lyase	27255834
BoPTAL1	ADE08261	*Brachypodium distachyon*	bifunctional ammonia-lyase	27255834
BoPAL3	ACN62413	*Brachypodium distachyon*	phenylalanine ammonia-lyase	27255834
BoPAL4	ADB97626	*Brachypodium distachyon*	phenylalanine ammonia-lyase	27255834
AsPAL1	GQ373155	*Avena strigosa*	phenylalanine ammonia-lyase	20459868

Table S7. Oligonucleotides used in this work.

SAD1, Segment 1	**AmyStaF1**	**ACGAGTGCTTGTTTTCCTCGTA**
	**Amy610R**	**CATACCGACAACCATATTTTTCCCC**
	Amy10F	GTATGGATTTCGTACCGTAAAT
	Amy16F	CAGTGGGATTCTCTTCATTATGC
	Amy18F	GTGGCTCATCACATTGATCACA
	Amy26R	ATCGTCTGCTTGTAGAGAGGA
	Amy25R	AGTTCAACCAAGATTTTAGACAAC
SAD1, Segment 2	**Amy610F**	**GTCGCTACATTTACAATCAACAGGCAT**
	**Amy109R**	**TCTATACCAACCTGTGCCTTCATTCC**
	Amy11F	CTGCGACAAGACCTCTATA
	Amy24R	GTCCCAATTAATGTTGCAGTAAG
	Sad1-M1453-S2-R2	CGTGGGTAATGAAGGTCCTCCTG
SAD1, Segment3	**Amy109F**	**TATCCATTATGACGACGAATCAACC**
	**Amy23R**	**TAGTCCACGACAATGTTCCGA**
	Amy05F	TGGATGTCATAGCTGGGA
	Amy06F	TATCCGCTGACCTTGTTG
	Amy17R	AGCCTTTTGATCTGTGGCGATA
	Amy06F-12F	ACGGACATTCGCTTGGTTAGAGG
	Sad1-M1673R	CAATGGCATCATACAACCTGTC
SAD1, Segment 4	**Amy12F**	**CTCAACCCTTCTGAGAGTTT**
	**AmyEndR1**	**TCAGCTCTTAATCGCAAGAAGT**
	Amy08F	GTGGATGGGGTGAAGACT
	Amy19F	TGGGCAATGTTGGCTTTAATTT
	Amy22R	TGGACACGGTCATCAAAACTG
	Sad1-M1233R1	GCATGGCTTATAGAACTACT
SAD2	**Sad2R1-2**	**CATCCGTGCAAATAGGTGCT**
	**Sad2F1**	**CTGCCAGTGTGAATCCTCTAGTC**
	Sad2F2	TCATGCCTGTACCATTCTAGC
	Sad2F3	CGTGGTAAATTGGAATGGCTG
	Sad2R2-2	GGTCCAGGAGGCAGTGATTGTACT
	Sad2R3	CACAAGCTAGAATGGTACAGGCA
	Sad2F4	TCAAAGAGGCAGGTCGGATG
SAD7	**SAD7 R**	**TTAGAGCTTTCCTGCGGGTG**
	**SAD7 F**	**ATGGAGAAGCTGCTCGTGGTG**
	Sad7-20-F	GTGGTGCTGCTGCTAGTGAC
	Sad7_q3'	GATCCATCTTCGGACCATGT
	sad7594F	ACTCCTTCTCCGGCTACACC
	Sad7-490-R	TCCACTTCTGGAGGAACACC
	Sad7-902F	TCACGACGCCTTCTTCTTCT
	Sad7-M456-G536-R	GATAAGGCGCTCTGGGTGAGAAGT
SAD9	**Sad9-1-5'**	**ATGGGGCATGTCCACACTAC**
	**Sad9_EndRs**	**CAATGATAGATCGAAATCCCAA**
	Sad9-726-F	GGGGATGCGTTTCAGTACAT
	SAD9G-19-F	CAAGGCGATGATGAGCACG
	Sad9-490-F	GGAGATGACAGCCAAGAAGG
	Sad9-473-R	CCTTCTTGGCTGTCATCTCC
CYP94D65	**AsC23-Seq-F1**	**GCAGGAGGAGGCTGAAGCAAAG**
	**AsC23-Seq-R1**	**GCTAGATGCCCGATGGAGGTTG**

	AsC23-Seq-F2	TGATCCTCAAGGTGCTGGGCCTG
	AsC23-Seq-F3	GAGGCAGAGCGTGGAGTTGATG
	AsC23-Seq-F4	TCACCAACTTCATACTCGCCGG
	AsC23-Seq-F5	TGGACGCGGAGACAGGGGTGTTC
	AsC23-Seq-R1	GCTAGATGCCCGATGGAGGTTG
	AsC23-Seq-R2	TCTAGGTGCATCGGCAAGCCGC
	AsC23-Seq-R3	TGATGGCGGCGTGGATGTAGTG
	AsC23-Seq-R4	CGGAGACACGAACCTGGACGAC
CYP72A476	**AsC30-Seq-F1**	**GGCCCAAGATCGGTCGAAAACAAC**
	**AsC30-Seq-R1**	**TATCAGATCCCGATTCATCCGCG**
	AsC30-Seq-F2	CGCGTCCTCCCGAACATCTGCAAC
	AsC30-Seq-F3	GGTAAGGACATGGAGGGAGTGC
	AsC30-Seq-F4	AGAGTCCAGCCCCAAGATGACG
	AsC30-Seq-F5	CCTATCCTGCTCATCCACCACG
	AsC30-Seq-R2	TCCCGTCGGCAAATCTCTCAGGC
	AsC30-Seq-R3	TGAGAAGTATGGACGTTGTGTCGG
	AsC30-Seq-R4	AGAGCGAGAAATGGCATCCCCG
	AsC30-Seq-R5	GCGAGGATCTCTTTCACGACGCC
SAD4	**UGT74a-F1**	**CGAACAAGAATCAAACACG**
	**UGT74a-R1**	**CTAGTTTCACGCTGAGTAC**
	UGT74a-F2	CAAACACGACCTCTCTC
	UGT74a-F3	TCTTGCGCCGTCTTCTC
	UGT74a-R2	CAAATCTTACTGTTCCACAG
	UGT74a-R3	CTTGTACGGGTTGGAGG
	UGT74a-R4	CCTTGTGGATCGTGCGGAAC
Segregation analysis	**GTa-933-F**	**CTTCTCCCTCTACTACCACCAC**
	**GTa-933-R**	**GTCCTGGGTGTTCGTCAACTC**
	GTa-933-seq	GACGCCCATCTTGAACTCCTC
Gateway primers	Sad4-Gateway-F	AAAAAGCAGGCTTC ATGGGCGAGGAGGCCGTAG
	Sad4-Gateway-R	AGAAAGCTGGGTC TTACTGTTCCACAGCCAC
	SAD10-Gateway-F	AAAAAGCAGGCTTC ATGGGGGCTGAGTGGGAG
	SAD10-Gateway-R	AGAAAGCTGGGTC TCATGCATCTAACCCCAC
	UGT74H7-Gateway-F	AAAAAGCAGGCTTC TGGGGGCTGAGCCGGGCCACGTA
	UGT74H7-Gateway-R	AGAAAGCTGGGTC CTAGATTTGTTTGGGCTGAGTTG
	PAL3-Gateway-F	AAAAAGCAGGCTTC ATGGCAGGCAACGGCCCCATC
	PAL3-Gateway-R	AGAAAGCTGGGTC CTAGACGTTGATGGGCAGGGG
	PAL2-Gateway-F	AAAAAGCAGGCTTC ATGGAGTGCGAGGGCTCCTTC
	PAL2-Gateway-R	AGAAAGCTGGGTC TCAGCAGAGCGGCAGGGGCTC

Primers in bold were used for amplification, and the following primers were used to sequence the corresponding fragment. Primers for SAD3 and SAD6 mutants were reported (23, 24).
